# Crosstalk Between Skeletal Muscle and Proximal Connective Tissues in Lipid Dysregulation in Obesity and Type 2 Diabetes

**DOI:** 10.3390/metabo15090581

**Published:** 2025-08-30

**Authors:** Nataša Pollak, Efua Gyakye Janežič, Žiga Šink, Chiedozie Kenneth Ugwoke

**Affiliations:** Institute of Anatomy, Faculty of Medicine, University of Ljubljana, 1000 Ljubljana, Slovenia; natasa.pollak@mf.uni-lj.si (N.P.); efua.ewusi-brown@mf.uni-lj.si (E.G.J.); ziga.sink@mf.uni-lj.si (Ž.Š.)

**Keywords:** lipid metabolism, inter-tissue crosstalk, skeletal muscle, adipose tissue, fascia, bone, myokines, adipokines, obesity, type 2 diabetes mellitus

## Abstract

**Background/Objectives:** Obesity and type 2 diabetes mellitus (T2DM) profoundly disrupt lipid metabolism within local microenvironments of skeletal muscle and its associated connective tissues, including adipose tissue, bone, and fascia. However, the role of local communication between skeletal muscle and its proximal connective tissues in propagating metabolic dysfunction is incompletely understood. This narrative review synthesizes current evidence on these local metabolic interactions, highlighting novel insights and existing gaps. **Methods:** We conducted a comprehensive literature analysis of primary research published in the last decade, sourced from PubMed, Web of Science, and ScienceDirect. Studies were selected for relevance to skeletal muscle, adipose tissue, fascia, and bone lipid metabolism in the context of obesity and T2DM, with emphasis on molecular, cellular, and paracrine mechanisms of local crosstalk. Findings were organized into thematic sections addressing physiological regulation, pathological remodeling, and inter-organ signaling pathways. **Results:** Our synthesis reveals that local lipid dysregulation in obesity and T2DM involves altered fatty acid transporter dynamics, mitochondrial overload, fibro-adipogenic remodeling, and compartment-specific adipose tissue dysfunction. Crosstalk via myokines, adipokines, osteokines, bioactive lipids, and exosomal miRNAs integrates metabolic responses across these tissues, amplifying insulin resistance and lipotoxic stress. Emerging evidence highlights the underappreciated roles of fascia and marrow adipocytes in regional lipid handling. **Conclusions:** Collectively, these insights underscore the pivotal role of inter-tissue crosstalk among skeletal muscle, adipose tissue, bone, and fascia in orchestrating lipid-induced insulin resistance, and highlight the need for integrative strategies that target this multicompartmental network to mitigate metabolic dysfunction in obesity and T2DM.

## 1. Introduction

Obesity and type 2 diabetes mellitus (T2DM) are among the most pressing global public health challenges of the 21st century [1,2,3], and are characterized by profound disturbances in systemic lipid metabolism and insulin action. Insulin resistance provides a unifying pathophysiological context linking these conditions, shaping how substrates are handled within and across tissues. Within this insulin-resistant milieu, these metabolic diseases not only drive excess adiposity but also lead to ectopic lipid accumulation in non-adipose tissues such as skeletal muscle, bone, and local connective tissues, promoting tissue dysfunction and further exacerbating metabolic derangements [4,5]. Increasing evidence indicates that beyond classical systemic mechanisms, local crosstalk among skeletal muscle, various adipose depots, fascia, and bone plays a crucial role in orchestrating lipid partitioning, insulin sensitivity, and tissue remodeling in obesity and T2DM [4,6,7].

Connective tissues, broadly defined by their mesenchymal origin, extracellular matrix content, and integrative structural roles, encompass diverse compartments including skeletal muscle endomysium and perimysium, adipose tissue, bone, and fascia [8]. These tissues not only provide mechanical support but also serve as metabolically active units with specialized capacities for lipid storage, oxidation, and intercellular signaling [4,9]. Skeletal muscle, comprising nearly 40% of adult body mass, is a principal site of insulin-stimulated glucose uptake and a dynamic regulator of lipid oxidation, directly influencing systemic energy balance [10,11]. Adipose tissue, while traditionally viewed as the primary lipid reservoir, exhibits remarkable depot- and cell-type-specific heterogeneity that shapes both local and systemic lipid homeostasis. Meanwhile, emerging studies reveal that bone and fascia are not merely passive scaffolds but actively participate in lipid metabolic processes through marrow adipocytes and fibro-adipogenic progenitors, respectively, adding further complexity to inter-tissue metabolic networks [12,13,14].

Despite substantial advances in understanding isolated tissue responses to metabolic overload, there remains limited integration of how these anatomically proximate yet functionally distinct connective tissues coordinate lipid handling under physiological conditions and become dysregulated in metabolic disease. This gap is particularly relevant given the increasing recognition of tissue-resident lipid intermediates—such as diacylglycerols, ceramides, and acylcarnitines—as local mediators of insulin resistance, inflammatory remodeling, and fibrogenesis [14,15,16]. Furthermore, new spatial omics technologies and imaging approaches underscore the heterogeneity of lipid accumulation and signaling across the muscle–adipose–bone–fascia axis, reinforcing the need for a cross-compartmental perspective [17,18,19].

Given these insights, the present narrative review aims to provide a comprehensive synthesis of current knowledge on the physiological regulation and pathological disruption of lipid metabolism within skeletal muscle and its contiguous connective tissues—specifically adipose depots, fascia, and bone—in the context of obesity and T2DM. These tissues were selected for detailed consideration due to their integral roles in mechanical force transmission and metabolic buffering, and emerging evidence highlighting their bidirectional metabolic crosstalk that underpins local insulin sensitivity and systemic lipid partitioning. Their inclusion moves beyond the traditional liver-muscle-adipose axis to provide a more focused perspective on the metabolic dialogue shaping musculoskeletal and connective tissue adaptations in obesity and T2DM. Understanding these intricate interactions is critical not only for elucidating the pathogenesis of insulin resistance and related complications but also for identifying novel tissue- or depot-specific therapeutic targets.

We performed a targeted search of PubMed, Web of Science, and ScienceDirect for January 2015–June 2025, with older studies included only when essential for foundational context or when uniquely informative. We considered peer-reviewed primary research articles in English only and included clinical (interventional/observational, imaging/biobank), epidemiological, in vivo animal, and in vitro/ex vivo mechanistic studies that examined lipid metabolism and/or crosstalk between skeletal muscle and at least one proximal connective tissue (adipose depots, bone/bone marrow adipose tissue, fascia) in obesity or T2DM. We define primary research as original human or experimental studies reporting new data; secondary syntheses (reviews, meta-analyses) and methodological commentaries were not considered primary. We excluded non-peer-reviewed sources (e.g., preprints, theses, conference abstracts), studies without a mechanistic link to muscle–connective tissue interactions, and pediatric-only studies unless mechanistically generalizable to adult disease. Searches combined controlled vocabulary and free-text terms targeting the concepts above (e.g., MeSH: “Skeletal Muscle,” “Adipose Tissue,” “Insulin Resistance,” “Diabetes Mellitus, Type 2”; keywords: lipid*, lipotoxic*, ceramide*, diacylglycerol*, myokin*, adipokin*, osteokin*, exosome*, “extracellular vesicle”, crosstalk/inter-organ/inter-tissue), with database-specific Boolean syntax (example PubMed string: “skeletal muscle” [MeSH] AND (adipose OR fascia OR “bone marrow adipose”) AND (lipid* OR lipotoxic* OR ceramide* OR diacylglycerol*) AND (“type 2 diabetes” OR obesity)). In addition to database queries, we performed backward and forward citation tracking of included records and limited manual searches of reference lists from key articles and recent reviews to identify any additional eligible studies. Screening of titles/abstracts and full texts was performed independently by two reviewers, with disagreements resolved by discussion (no blinding).

This review is structured into three main sections. First, we outline physiological lipid metabolism regulation at the skeletal muscle–connective tissue interface, highlighting the roles of myocellular lipid droplets, adipocyte subtypes, bone marrow lipid reservoirs, and fascia-resident progenitor cells in energy homeostasis. Next, we discuss disruptions to these regulatory processes caused by obesity and T2DM, emphasizing mechanisms of lipid-induced damage, including ectopic lipid accumulation, mitochondrial dysfunction, fibrosis, and inflammatory remodeling. Finally, we examine emerging insights into local tissue crosstalk mediated by cytokines, bioactive lipids, exosomes, and extracellular matrix signaling, which perpetuate lipid dysregulation and insulin resistance at this interface.

## 2. Physiological Regulation of Lipid Metabolism at the Skeletal Muscle–Connective Tissue Interface

### 2.1. Healthy Skeletal Muscle Lipid Uptake, Oxidation, and Storage

In healthy skeletal muscle, lipid metabolism is a crucial process for maintaining energy homeostasis, particularly during fasting or prolonged physical activity. Recent studies demonstrate that skeletal muscle adapts to fasting by preserving mitochondrial oxidative capacity and increasing lipid storage and utilization, enabling sustained performance [20,21]. Skeletal muscle is a metabolically active tissue that plays a central role in lipid and glucose utilization at rest and during activity, supported by its high mitochondrial content and substrate transport capacity [21,22]. During submaximal or prolonged activity, mitochondrial oxidative phosphorylation becomes the predominant source of adenosine triphosphate (ATP), utilizing both carbohydrates and fats depending on substrate availability and physiological demands. This metabolic shift is tightly regulated by exercise, fasting, and hormonal cues that govern substrate selection and mitochondrial flux [21]. Skeletal muscle comprises metabolically distinct fiber types that differ in myosin heavy chain isoforms, mitochondrial content, and substrate preference, with oxidative type I and type IIa fibers showing higher fatty-acid oxidation capacity and glycolytic type IIx fibers favoring rapid force generation and glycolytic ATP production. In adult human limb skeletal muscle, the type IIb (MYH4) isoform is not expressed [23,24].

Fatty acid (FA) uptake into skeletal muscle follows a two-step process: first, trans-endothelial transport regulated by cluster of differentiation 36 (CD36) and fatty acid transport protein 4 (FATP4) located on endothelial cells, which is enhanced by 3-hydroxyisobutyrate (3-HIB)—a valine catabolite secreted downstream of peroxisome proliferator-activated receptor gamma coactivator 1-alpha (PGC1α) activation [25]—and vascular endothelial growth factor B (VEGF-B) signaling that induces endothelial FATP expression [26]. Once across the endothelium, FAs enter the myocyte via CD36, plasma membrane fatty acid-binding protein (FABPpm), and various FATPs, where they are either directed toward mitochondrial oxidation or stored in lipid droplets (LDs). These LDs are especially abundant in oxidative fibers and interact with mitochondria through perilipin 5 (PLIN5)-enriched tethering sites that facilitate FA transfer [27,28]. In glycolytic fibers, PLIN2-coated LDs dominate and may buffer lipids to prevent lipotoxicity while indirectly influencing muscle structure—loss of PLIN2 reduces lipid storage but paradoxically induces hypertrophy [29].

The abundance and localization of transporters adapt to the energetic state. Under physiological conditions, CD36, FATP1, FATP4, and FABPpm translocate from intracellular compartments to the sarcolemma in response to energetic stimuli—such as muscle contraction, insulin, or lipid oversupply—thereby limiting long-chain fatty acid (LCFA) uptake to periods of increased metabolic demand. CD36 translocation is mediated by insulin- and contraction-stimulated signaling involving Rab guanosine triphosphatase (Rab GTPase)/exocyst complex machinery, as demonstrated in murine muscle cells [30]. In vivo and ex vivo models further show that CD36 is essential for fatty acid oxidation during exercise: CD36-knockout mice exhibit impaired LCFA oxidation both at the whole-body level and in isolated contracting skeletal muscle [28]. Similarly, FATP4 (solute carrier family 27 member 4 [SLC27A4]) translocation is regulated by TBC1 domain family member 1 (TBC1D1)/TBC1D4 Rab GTPase-activating proteins (RabGAPs), promoting sarcolemmal fatty acid transport during insulin or contraction stimuli [31]. Training alters transporter levels: FATP4 increases and correlates positively with lipid oxidation, while FATP1 decreases [28], preferentially directs lipids toward oxidation, whereas FATP1 may buffer or partition fatty acids based on metabolic demand.

The fate of internalized FAs—storage versus oxidation—depends on dynamic interactions between lipid droplets and mitochondria, as well as systemic cues. Inside the muscle, FAs are oxidized in mitochondria or stored as triacylglycerols (TAGs). Diacylglycerol O-acyltransferase 2 (DGAT2) drives de novo lipogenesis, while DGAT1 is implicated in FA uptake without influencing oxidation [32]. In endurance-trained athletes, type I fibers exhibit 2–3-fold higher abundance of adipose triglyceride lipase (ATGL), hormone-sensitive lipase (HSL) and PLIN5 compared to type II fibers [33], enabling efficient LD-mitochondria coupling and maximal fat oxidation.

### 2.2. Lipid Droplet Dynamics in Skeletal Muscle

Lipid droplets in healthy skeletal muscle are highly dynamic organelles that undergo continuous remodeling to balance storage and mobilization. These structures exhibit fiber-type-specific organization, with type I fibers containing larger, more stable lipid droplets that interact with mitochondria through PLIN5-enriched contact sites [27]. PLIN5 serves as a critical regulator of these interactions, creating specialized microdomains that facilitate direct fatty acid transfer to mitochondria during energy demands [28,34]. PLIN5 not only tethers LDs to mitochondria but also recruits ATGL and HSL to droplet surfaces, creating localized lipolytic ‘hotspots’ during exercise [35]. This spatial coordination ensures rapid FA channeling to mitochondria while preventing lipotoxic intermediates. LD size is dynamically controlled by fusion/fission events, with larger droplets in type I fibers favoring sustained energy release, while smaller droplets in type II fibers support rapid mobilization [36]. The turnover of lipid droplets involves coordinated action of lipogenic and lipolytic enzymes—DGAT2 drives de novo lipid droplet expansion while ATGL, activated by its cofactor comparative gene identification-58 (CGI-58), initiates TAG hydrolysis [32,37]. Notably, CGI-58 localizes to both lipid droplets and mitochondria, suggesting a role in spatially coordinating lipolysis with oxidative capacity [37]. This organization helps explain the “athlete’s paradox” in which elevated intramyocellular lipid (IMCL) coexists with insulin sensitivity: when LD–mitochondria coupling and proteolytic control are optimal, lipid stores become readily oxidizable fuel rather than lipotoxic burden [36,38].

For clarity, IMCL droplets within myofibers are distinct from intermuscular adipose tissue (IMAT), which consists of adipocytes between and around muscle fibers beneath the deep fascia. IMCL droplets act as a dynamic fuel reservoir whose metabolic impact depends on droplet size/location and coupling to mitochondria (e.g., PLIN-coated, intermyofibrillar pools). High IMCL can coexist with high insulin sensitivity in trained muscle (the “athlete’s paradox”) when droplet turnover and oxidative capacity are high [38]. IMAT is a distinct adipose depot and a robust negative correlate of insulin sensitivity and muscle quality in humans, independent of total adiposity in several cohorts [39]. Mechanistically, IMAT consistently associates with lower insulin sensitivity and poorer muscle quality in humans, independent of overall adiposity, and human studies suggest local paracrine effects on adjacent myofibers [39,40] (see Section 3.2 for a more detailed discussion of the metabolic significance of IMAT).

### 2.3. Regulation of Lipid Metabolism in Skeletal Muscle

Exercise plays a key role in dynamically regulating lipid mobilization in skeletal muscle. During moderate-intensity activity such as cycling, hormone-sensitive lipase (HSL) translocates to PLIN5-associated lipid droplets, marking in vivo activation of lipolysis [35]. This redistribution occurs preferentially to PLIN5-coated droplets, which are more metabolically active. PLIN5 thereby facilitates spatially focused lipolysis. While ATGL distribution remains stable during acute exercise, its activity at lipid droplets increases via post-translational modifications (e.g., phosphorylation), complementing HSL-mediated lipolysis. Chronic endurance training increases ATGL expression in human skeletal muscle [41], enhancing basal fat oxidation capacity through elevated lipolysis [42] and improved mitochondrial fatty acid utilization [28].

Lipid metabolism in skeletal muscle is also governed by endocrine and paracrine signals, particularly myokines and adipokines that regulate substrate use and systemic energy balance. Pro-lipolytic regulators such as interleukin-6 (IL-6), interleukin-15 (IL-15), fibroblast growth factor 21 (FGF21), irisin, and 12,13-diHOME stimulate fatty acid oxidation and mitochondrial gene expression via AMPK (AMP-activated protein kinase) and PGC-1α pathways [43,44,45,46,47,48]. In contrast, myostatin acts as an anti-lipolytic signal, promoting lipid accumulation and muscle aging through suppression of oxidative pathways [49]. Metabolic sensors including adiponectin, β-aminoisobutyric acid (BAIBA), and apelin modulate insulin sensitivity, fatty acid utilization, and mitochondrial activity [50,51,52]. IL-15 promotes lipid utilization through peroxisome proliferator-activated receptor delta (PPARδ) activation, linking muscle metabolism to whole-body energy partitioning [44].

Endocrine and paracrine signals modulate skeletal-muscle lipid use by converging on AMPK/PGC-1α-dependent transcriptional programs and acute lipolytic control. Skeletal muscle integrates lipid availability with contractile and nutrient state through conserved nutrient-sensing pathways. AMPK (AMP-activated protein kinase) responds to energetic stress (↑AMP/ADP:ATP) and acutely promotes fatty-acid oxidation by phosphorylating acetyl-CoA carboxylase 2 (ACC2), lowering malonyl-CoA and disinhibiting CPT1-mediated mitochondrial import; it also supports oxidative remodeling via PGC-1α–dependent programs and restrains mTORC1 (mechanistic target of rapamycin complex 1) signaling during low energy. These actions collectively favor lipid catabolism over storage during exercise and fasting [53,54]. By contrast, mTORC1 integrates insulin/ insulin-like growth factor 1 (IGF-1)and amino-acid sufficiency to promote protein synthesis and lipogenesis (in part via SREBP), while suppressing autophagy. Chronic mTORC1 hyperactivation in skeletal muscle has been linked to impaired oxidative metabolism and a shift toward glycolytic reliance, with emerging genetic models showing altered lipidomic responses to exercise and increased intramuscular triglycerides under constitutive mTORC1 activity [55]. At the transcriptional level, PPARδ (PPAR-β/δ) is highly expressed in muscle and coordinates oxidative phenotype by upregulating genes for fatty-acid uptake and β-oxidation (e.g., CPT1B, PDK4, FABP3), enhancing mitochondrial capacity and insulin sensitivity; exercise-induced cues such as BDNF can engage PPARδ-dependent reprogramming during recovery.

Together, the AMPK–mTOR–PPARδ axis provides a mechanistic scaffold linking nutrient state and contraction to lipid partitioning—toward oxidation under energy stress (AMPK/PPARδ) and toward storage/anabolism with prolonged nutrient/anabolic signaling (mTORC1) [56,57]. In vivo, these pathways cooperate with transporter translocation (CD36/FATPs/FABPpm) and LD remodeling (ATGL/HSL/PLIN5) to align FA entry, mobilization, and oxidation with energetic demand, providing a mechanistic bridge between acute exercise physiology and chronic training adaptations. These tightly coordinated mechanisms highlight the metabolic flexibility of healthy skeletal muscle and underscore the critical roles of mitochondrial function, lipid transporters, and hormonal regulation in energy homeostasis.

### 2.4. Adipose Tissue Depots and Adipocyte Phenotypes in Physiological Lipid Metabolism

Adipose tissue functions as both an energy reservoir and an endocrine organ that shapes systemic and local metabolism through depot- and cell-type-specific programs [58,59]. It is distributed across multiple anatomical depots, most prominently subcutaneous, visceral, and intermuscular fat, each with distinct metabolic profiles and regulatory roles [60]. Within these depots reside different adipocyte subtypes, including classical white, thermogenic brown, and inducible beige adipocytes, which together orchestrate lipid storage, mobilization, and oxidation to maintain metabolic homeostasis [58]. The functional diversity of these adipocyte populations underlies their capacity to buffer circulating lipids and modulate insulin sensitivity, processes that are compromised in obesity and T2DM.

White adipose tissue (WAT) is widely distributed across the body in depots such as subcutaneous (SAT), visceral (VAT), and mammary adipose tissue, and serves as the principal site for lipid storage, mobilization, and endocrine signaling. Comprising mature adipocytes interspersed with stromal cells, immune cells, and vasculature, WAT secretes adipokines including leptin and adiponectin, which regulate insulin sensitivity and lipid metabolism in nearby tissues, notably skeletal muscle [61]. WAT displays remarkable physiological plasticity, with cold exposure or β3-adrenergic stimulation promoting catabolic remodeling and the emergence of metabolically active adipocyte subpopulations [58].

Brown adipose tissue (BAT), derived developmentally from myogenic precursors, contains multilocular lipid droplets and mitochondria rich in uncoupling protein 1 (UCP1), enabling non-shivering thermogenesis [58,62]. Cold exposure enhances BAT activity through sympathetic stimulation and increases secretion of batokines such as FGF21, which amplifies fatty acid oxidation and mitochondrial uncoupling [62,63,64]. These mechanisms contribute to systemic energy expenditure, but also influence local lipid turnover and may indirectly protect skeletal muscle from lipotoxic stress under metabolic challenge [65].

Beige adipocytes are inducible thermogenic cells embedded within WAT, characterized by multilocular lipid droplets and UCP1-expressing mitochondria—a process termed “browning.” Chronic cold exposure, β3-adrenergic agonists, or exercise promotes their emergence either via transdifferentiation of white adipocytes or de novo differentiation from resident precursors [58,66]. Beige adipocytes enhance glucose and lipid uptake, reduce insulin demand, and increase energy expenditure, representing promising therapeutic targets for obesity and T2DM [67]. Notably, inhibition of autophagy pathways involving Atg5 or Atg12 impedes beige-to-white conversion, underscoring the dynamic regulation of this phenotype [68].

WAT primarily stores energy, whereas thermogenic adipocytes dissipate energy through UCP1-dependent proton leak coupled to high oxidative metabolism. The transcriptional regulator PRDM16, together with PPARγ and PGC-1α, drives thermogenic gene programs, while adrenergic signaling provides rapid control of lipolysis and heat production. In humans, sympathetic regulation of brown/thermogenic fat shows species-specific features: β2-adrenergic input is the dominant driver of thermogenesis in brown adipocytes, and in vivo responses to β3-agonists such as mirabegron are heterogeneous [69,70,71]. Cold exposure and selective pharmacology recruit thermogenic fat in the supraclavicular/neck region of adults, and the presence of active BAT associates with favorable cardiometabolic profiles at the population level [72,73]. However, the extent and durability of beige remodeling within classical subcutaneous WAT in adults, and the magnitude of its contribution to whole-body energy expenditure, remain uncertain, reflecting mixed pharmacologic results and clear differences from rodent β3-adrenergic biology.

### 2.5. Physiological Roles of Bone in Lipid Metabolism

Beyond structural and hematopoietic roles, bone acts as an endocrine regulator of systemic energy and lipid metabolism. Osteoblast-derived osteocalcin in its undercarboxylated form enhances insulin secretion, promotes β-cell proliferation, and improves peripheral insulin sensitivity, thereby influencing lipid use and storage [74,75]. Osteocytes produce sclerostin, a Wnt pathway modulator that links mechanical loading to adipogenesis; its suppression appears to increase energy expenditure and reduce fat mass [76,77]. These endocrine signals position bone as a coordinator of lipid handling across skeletal muscle, adipose tissue, and liver. Preclinical studies identify undercarboxylated osteocalcin as a regulator of insulin secretion and sensitivity. Human findings are mixed: several cohorts report inverse associations with dysglycemia and incident diabetes, whereas others show null or assay-dependent results; randomized trials remain scarce. Newer assays that quantify bioactive or undercarboxylated osteocalcin strengthen associations with insulin resistance and β-cell function but do not establish causality [78,79]. Accordingly, we treat osteocalcin as a biomarker of connective tissue crosstalk with suggestive, not definitive, metabolic effects in humans.

Moreover, bone marrow itself constitutes a dynamic lipid reservoir comprising marrow adipocytes, which account for up to 70% of adult marrow volume. These adipocytes are phenotypically distinct from peripheral white adipocytes, exhibiting unique transcriptomic signatures and responding to systemic metabolic cues by modulating lipolysis and fatty acid release [80]. Marrow lipids provide substrates for local osteoblast and osteoclast energy needs, particularly under conditions of increased bone remodeling. Notably, bone marrow adiposity is inversely correlated with bone mineral density and may impact skeletal integrity through altered paracrine signaling, thereby linking lipid metabolism to bone strength and overall metabolic health [12,80].

Bone marrow adipose tissue (BMAT) is molecularly distinct from white and brown adipose tissue, with a transcriptome indicating altered glucose handling and reduced insulin responsiveness. BMAT exists as regulated (rBMAT) and constitutive (cBMAT) subtypes that differ in anatomic distribution, plasticity, and responsiveness to environmental cues (e.g., cold, overnutrition) [81,82]. BMAT secretes adipokines (e.g., adiponectin, leptin) and cytokines that can influence bone remodeling, hematopoiesis, and whole-body metabolism; adipokine output varies with BMAT subtype and metabolic state. In parallel, leptin integrates peripheral energy status with bone through central neuroendocrine pathways, including hypothalamic–sympathetic signals that modulate bone turnover and may secondarily affect marrow adiposity. Human and translational evidence supports leptin’s dual direct and central actions on bone, while indicating context-dependence in obesity and diabetes [82,83].

### 2.6. Emerging Role of Fascia in Local Adipogenesis and Lipid Metabolic Regulation

Fascia, a mesoderm-derived connective tissue traditionally viewed as a structural matrix surrounding muscles and organs, is now recognized to harbor multipotent progenitors capable of adipogenic, osteogenic, and chondrogenic differentiation in vitro [84,85,86,87]. The fascia tissue contains several cell types including fibroblasts and adipocyte clusters, and an extensive extracellular matrix enriched in collagen and hyaluronan. In humans, the superficial fascia separates superficial and deep subcutaneous adipose compartments and is richly innervated, with nerve fibers concentrated around blood vessels and adjacent to adipocytes, suggesting a potential neurovascular interface that can sense mechanical and metabolic cues [88]. Human studies also identify a lymphatic plexus within the superficial fascia, suggesting a role in interstitial fluid and immune signaling [89]. Mast cells are present within human superficial fascia and localize near nerves and vessels, consistent with immuno-metabolic crosstalk [90]. Given these structural and cellular features, we consider fascia a metabolically responsive interface connecting subcutaneous depots and muscle, while acknowledging that direct lipid-handling functions in humans remain to be demonstrated.

Previous work from rat models revealed that the superficial fascia contains not only fibroblasts and mast cells but also abundant lineage-committed preadipocytes, particularly aligned along vascular structures, actively participating in adipogenesis during developmental periods [86,87]. Fibro-adipogenic progenitors (FAPs) are mesenchymal stromal cells that reside in the endomysial–perimysial interstitium and support myogenesis while retaining adipogenic and fibrogenic potential. In mice, they are typically isolated as platelet-derived growth factor receptor alpha-positive (PDGFRα+)/Sca1^+^/CD34^+^ stromal cells; in humans, Sca1 lacks a direct ortholog and FAPs are identified by PDGFRα^+^/CD34^+^/THY1^+^ phenotypes with additional heterogeneity, including an MME^+^ subset that exhibits strong adipogenic potential during fatty infiltration [91,92,93].

Mast cell-derived heparin within fascia was shown to act as an endogenous trigger for adipocyte formation [86]. Notably, fascia-derived progenitors exhibit substantial heterogeneity across species and anatomical sites, with superficial fascia of rats showing high adipogenic potential, whereas visceral fasciae displayed minimal adipogenesis, possibly due to sparse vascularization [85,87]. Consistent with this, in muscle interstitial fascia PDGFRα^+^ FAPs differentiate into adipocytes under pathological or regenerative conditions, promoting local lipid accumulation and fibrosis [14,94]. Wnt signals constrain adipogenesis; in particular WNT7A suppresses FAP adipogenesis by engaging an alternative Wnt–Rho–YAP/TAZ route that operates largely independently of β-catenin, and pharmacologic GSK3 blockade stabilizes β-catenin and reduces adipogenesis in large-animal FAPs. Notch signaling functions as a context-dependent brake on mesenchymal adipogenesis in muscle and has emerged as a regulator of stromal fate decisions in the muscle niche [95,96,97].

Importantly, fascia-derived adipocytes exhibit distinct functional phenotypes, maintaining high basal lipolysis yet demonstrating reduced catecholamine sensitivity compared to classic subcutaneous and visceral adipocytes, highlighting fascia as a potential unique metabolic niche for local lipid turnover [84,87]. This adds a new dimension to understanding regional lipid storage and mobilization, with possible consequences for local muscle-fascia-adipose crosstalk in obesity and T2DM.

Recent advances have led to the successful generation of functional 3D fat organoids from rat superficial fascia fragments, which recapitulate key histological and metabolic properties of adipose tissue, including unilocular lipid storage, triglyceride hydrolysis, and adipokine secretion [87]. Cells outgrown from fascia in fibrin hydrogels expressed markers of adipogenic stromal progenitors (e.g., CD29, CD90, CD106, CD44) but low levels of endothelial and hematopoietic markers, reflecting their tissue-specific niche. These organoids support the concept that adipocytes can arise from non-adipose fascia, providing a novel perspective on the local origins of adipose tissue [87]. Collectively, these findings highlight fascia not merely as a passive structural scaffold but as a dynamic participant in local adipose formation and lipid metabolism, potentially modulating the regional balance between lipid storage, mobilization, and muscle insulin sensitivity. This emerging role underscores the importance of considering fascial contributions in studies of muscle-adipose crosstalk and metabolic dysregulation in obesity and T2DM.

## 3. Lipid Dysregulation in Skeletal Muscle and Local Connective Tissues in Obesity and T2DM

### 3.1. Pathological Alterations in Skeletal Muscle Lipid Handling in Obesity and T2DM

**Figure 1:**
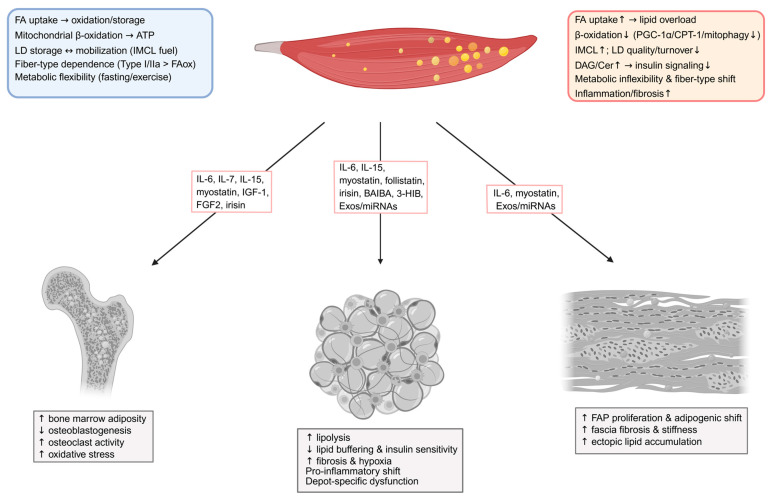
Skeletal muscle pathophysiology and crosstalk with proximal connective tissues in obesity and type 2 diabetes. Schematic overview of physiological skeletal-muscle lipid handling (blue) contrasted with dysregulation in obesity and type 2 diabetes (orange), highlighting representative muscle-associated mediators to adjacent bone tissue, adipose tissue and fascia, and the typical alterations observed in each target tissue. Tissue layout and lipid relevant constituents: skeletal muscle (top; metabolically distinct fiber types with intracellular and extracellular lipid pools), bone (bottom left; bone cells and bone-marrow adipocytes), adipose (bottom middle; white, beige, and brown adipocytes with stromal–immune components), and fascia (bottom right; collagen-rich extracellular matrix, fibroblasts, and FAPs). In physiological conditions, skeletal muscle couples fatty-acid uptake with mitochondrial β-oxidation and flexible lipid-droplet storage–mobilization to sustain ATP supply across fiber types. In obesity/T2DM, fatty-acid influx outpaces oxidative capacity with reduced β-oxidation/mitophagy and low LD turnover, yielding IMCL accumulation, DAG/ceramide build-up, impaired insulin signaling, metabolic inflexibility, and pro-inflammatory/fibrotic remodeling. Muscle-derived or muscle-associated mediators are depicted along arrows indicating directional local signaling to bone, adipose tissue, and fascia. These muscle-derived cues collectively modulate lipid handling, inflammatory tone, and matrix remodeling across these tissues, shifting local homeostasis toward dysfunction under obesity/T2DM. Abbreviations: 3-HIB, 3-hydroxyisobutyrate; ATP, adenosine triphosphate; BAIBA, β-aminoisobutyric acid; Cer, ceramide; CPT1, carnitine palmito-yltransferase-1; DAG, diacylglycerol; Exos, exsosomes; FA, fatty acid; FAox, fatty-acid oxidation; FAPs, fibro-adipogenic progenitors; FGF2, fi-broblast growth factor-2; IGF-1, insulin-like growth factor-1; IL, interleukin; IMCL, intramyocellular lipid; LD, lipid droplet; miRNA, microRNA; PGC-1α, peroxisome proliferator-activated receptor-γ coactivator-1α.

Obesity and T2DM lead to profound disturbances in skeletal muscle lipid metabolism, manifesting as excessive fatty acid (FA) uptake, mitochondrial overload, defective lipid droplet (LD) regulation, and chronic inflammation. These changes impair muscle energy metabolism and disrupt systemic metabolic homeostasis. Figure 1 summarizes muscle lipid metabolism in health versus obesity/T2DM and highlights muscle-to-proximal connective tissue signaling.

Under healthy conditions, fatty acid transporters—CD36, FATP1/4, and FABPpm—are recruited to the sarcolemma in response to increased energy demand, ensuring tightly regulated FA entry. In obesity and T2DM, CD36 becomes constitutively localized at the membrane through S-acylation by zinc finger DHHC-type palmitoyltransferase 4 (zDHHC4), which is upregulated by forkhead box protein O1 (FoxO1) signaling [98]. Additional mechanisms such as PKCζ- and TBC1D1-mediated translocation also contribute to sarcolemmal retention of CD36 in response to lipid oversupply [99]. CD36 dynamics are further modulated through palmitoylation-regulated endocytosis, which is disrupted in insulin resistance, impairing CD36 internalization and enhancing FA uptake [100]. Elevated circulating fatty acids due to impaired insulin-mediated suppression of lipolysis in skeletal muscle and adipose tissue further exacerbates transporter dysregulation [101]. Therapeutically, inhibition of zDHHC4 or FoxO1 restores CD36 dynamics and improves insulin sensitivity [98].

Recent mechanistic insights highlight a critical role for the exocyst complex in regulating skeletal muscle lipid uptake through CD36 trafficking. In skeletal muscle cells, insulin and contraction stimulate the exocyst-dependent translocation of CD36 to the plasma membrane, directly increasing free fatty acid (FFA) uptake [30,102]. This mechanism parallels known exocyst control of GLUT4 trafficking, underscoring how convergent vesicular pathways govern both glucose and lipid transporter delivery. Importantly, chronic CD36 membrane localization, often seen in obesity, enhances FFA influx, promoting intramyocellular lipid accumulation that impairs insulin-stimulated GLUT4 translocation and exacerbates local insulin resistance. These findings extend the understanding of muscle lipid dysmetabolism by identifying exocyst-mediated CD36 trafficking as a pivotal node in the crosstalk between skeletal muscle lipid handling and insulin sensitivity.

Mitochondrial β-oxidation fails to compensate for lipid influx due to suppressed PGC-1α signaling, carnitine palmitoyltransferase 1 (CPT-1) inhibition [103,104,105], and defective mitophagy via BCL2/adenovirus E1B 19 kDa interacting protein 3 (BNIP3)/NIP3-like protein X (NIX) pathway impairment, as shown in muscle-specific BNIP3L knockout mice, which accumulate mitochondria and display enhanced insulin sensitivity alongside altered metabolic signaling [106]. Early defects in fatty acid oxidation and mitochondrial biogenesis have also been demonstrated in the progression of insulin resistance [104]. Surplus acyl-CoAs are diverted into lipotoxic intermediates: sn-1,2-diacylglycerols (DAGs) activate protein kinase C theta/epsilon (PKCθ/ε), disrupting insulin receptor substrate 1 (IRS-1)/phosphoinositide 3-kinase (PI3K)/protein kinase B (Akt) [107]. Ceramides impair insulin signaling in a species- and tissue-specific manner; in skeletal muscle, CerS1-derived C18:0 ceramides promote insulin resistance and are required for full glucose intolerance [108]. In contrast, muscle-specific deletion of CerS6, which generates C16:0 ceramides, does not confer metabolic protection [109], highlighting the dominant role of C18:0 ceramides in skeletal muscle pathology. Lipid excess also promotes reactive oxygen species (ROS)/endoplasmic reticulum (ER) stress via NADPH oxidase 2 (NOX2) activation and c-Jun N-terminal kinase (JNK) signaling [110,111]. Recent findings further demonstrate that a high-fat diet induces ER stress through activation of inositol-requiring enzyme 1 (IRE1), protein kinase RNA-like ER kinase (PERK), and activating transcription factor 6 (ATF6) pathways in skeletal muscle, while exercise alleviates these changes via AMPK/PGC-1α–mediated autophagy activation [112].

Healthy oxidative (type I) fibers store TAGs in PLIN5-coated LDs that are dynamically mobilized. In T2DM, PLIN5 degradation exposes LDs to ATGL/HSL, promoting uncontrolled lipolysis [35]. Lipophagy is suppressed due to reduced transcription factor EB (TFEB) activity [113,114], impairing turnover. The apolipoprotein L6 (ApoL6) protein has recently been identified as a lipid droplet–associated inhibitor of lipolysis, interfering with the PLIN1–HSL interaction and contributing to lipid droplet stability [115]. The paradox of high IMCL coexisting with insulin sensitivity in athletes reflects the importance of lipid droplet quality—composition, turnover, localization—rather than quantity [36,116]. Disruption of lipid droplet buffering capacity may also enhance cellular stress: lipid droplets sequester unfolded proteins and excess fatty acids during ER stress, and their dysfunction exacerbates lipotoxicity [117].

In obesity and T2DM, skeletal muscle shifts toward a fast glycolytic profile with more type IIx and fewer type I fibers. This remodeling coincides with reduced mitochondrial content and respiration, altered fission–fusion dynamics, and disrupted electron-transport supercomplexes [118,119]. The resulting constraints on fatty-acid oxidation favor the accumulation of lipotoxic intermediates and exacerbate insulin resistance. These compositional and organellar changes are consistent with downregulated AMPK [55,57,120] programs and relative mTORC1 predominance under chronic nutrient excess, which together reduce metabolic flexibility in T2DM (see also Section 2.2 and Section 2.3). Obesity-associated capillary rarefaction compounds the problem: reduced microvascular density limits fatty acid clearance and oxygen delivery, promoting ectopic lipid deposition and hypoxic stress at the fascia–muscle interface [121]. FAPs shift toward pathogenic phenotypes through TGF-β-mediated suppression of transcription factor 7-like 2 (TCF7L2)—a Wnt pathway transcription factor critical for maintaining progenitor quiescence. TGF-β drives TCF7L2 degradation via the ubiquitin-proteasome system while simultaneously repressing its transcription through histone deacetylases (HDACs), thereby promoting ECM deposition and pro-fibrotic signaling [122]. CD36 deficiency has been associated with paradoxical improvements in glucose uptake alongside impaired microvascular perfusion, highlighting its dual role in fatty acid transport and endothelial insulin responsiveness [123]. Beyond structural rarefaction, muscle-derived exosomes modulate endothelial function and drive angiogenic activity through ROS-mediated NF-κB rather than VEGF-dependent pathways [124].

Skeletal muscle also influences surrounding stromal and immune populations via its secretome. Since myostatin was first identified as a myokine in 1997, secretome-based analysis of human myocyte culture medium has revealed over 600 myokines to date [125]. Among these, IL-6, myostatin, FGF21, irisin, and IL-15 mediate paracrine crosstalk affecting endothelial cells, fibroblasts, adipocytes, and immune cells. IL-6 and IL-15 promote lipolysis and fatty acid oxidation via AMPK and PGC-1α signaling pathways [43,45]. FGF21 and irisin enhance mitochondrial gene expression and improve glucose and lipid metabolism [48,126]. Myostatin, by contrast, inhibits adipogenesis and promotes fibrosis [44]. This paracrine network becomes maladaptive under chronic lipid overload, reinforcing local inflammation, fibrogenesis, and metabolic inflexibility. Diabetic muscle loses the ability to switch between glucose and lipid oxidation due to chronic lipid overload, AMPK suppression [105,127], and impairment of the sirtuin 3 (SIRT3)–superoxide dismutase 2 (SOD2) axis [114]. Fascia–muscle interplay is also altered: PDGFRα+ mesenchymal progenitors within muscle-associated connective tissue drive ectopic adipogenesis [128].

### 3.2. Adipose Tissue Remodeling and Depot-Specific Dysfunction in Obesity and T2DM

**Figure 2:**
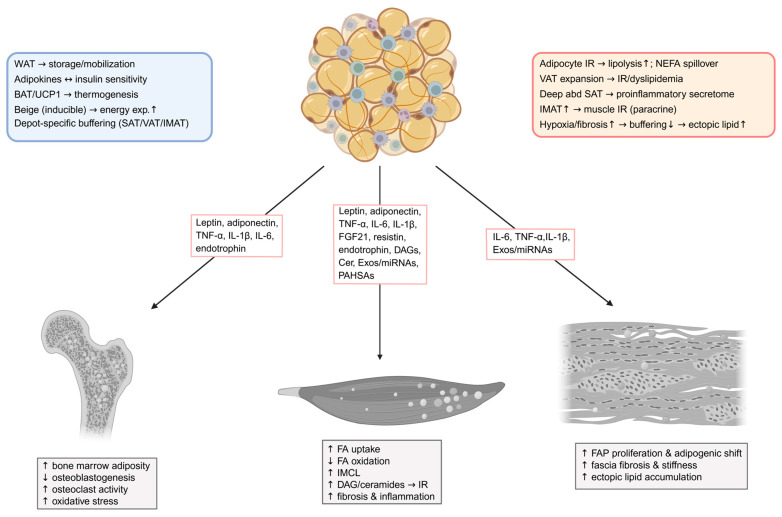
Adipose tissue dysregulation and crosstalk with proximal connective tissues in obesity and type 2 diabetes. Schematic overview of physiological adipose-tissue lipid handling (blue) versus dysregulation in obesity/T2DM (orange), with representative adipose-derived mediators directed to skeletal bone, muscle, and fascia and the corresponding alterations in each target tissue. Tissue layout and lipid-relevant constituents: adipose (top; white, beige, and brown adipocytes with depot-specific buffering across SAT/VAT/IMAT), bone (bottom left; bone cells and bone-marrow adipocytes), skeletal muscle (bottom middle; metabolically distinct fibers with intracellular and extracellular lipid pools), and fascia (bottom right; collagen-rich extracellular matrix, fibroblasts, and FAPs). Physiologically, WAT supports lipid storage/mobilization and adipokine-linked insulin sensitivity, BAT/UCP1 enables thermogenesis, beige adipocytes raise energy expenditure, and depots buffer lipid flux. In obesity/T2DM, adipocyte insulin resistance elevates lipolysis and NEFA spillover; VAT expands with dyslipidemia while deep abdominal SAT acquires a pro-inflammatory secretome; IMAT increases with paracrine effects on muscle insulin resistance; hypoxia/fibrosis reduce buffering and promote ectopic lipid deposition. Adipose-derived mediators are indicated along arrows to bone, muscle, and fascia, and target panels summarize the downstream outcomes. Abbreviations: BAT, brown adipose tissue; BAT/UCP1, bone marrow adipose tissue; Cer, ceramides; DAGs, diacylglycerols; Exos, exosomes; FA; fatty acid; FAPs, fibro-adipogenic progenitors.; FGF21, fibroblast growth factor-21; IL, interleukin; IL-1β, interleukin-1 beta; IMAT, intermuscular adipose tissue; IMCL, intramyocellular lipid; IR, insulin resistance; miRNA, microRNA; NEFA, non-esterified fatty acids; PAHSAs, palmitic acid esters of hydroxystearic acids; SAT, subcutaneous adipose tissue; TNF-α, tumor necrosis factor-alpha; UCP1, uncoupling protein-1; VAT, visceral adipose tissue; WAT, white adipose tissue.

In obesity and T2DM, adipose tissue undergoes profound functional remodeling, characterized by insulin resistance, altered lipid turnover, and chronic low-grade inflammation, all of which disrupt local lipid homeostasis and propagate metabolic stress to adjacent skeletal muscle and connective tissues. Figure 2 summarizes physiological and pathological features of adipose depots and the adipose-derived mediators that communicate with skeletal muscle, bone tissue, and fascia.

Subcutaneous adipose tissue (SAT) is the largest and most anatomically diverse adipose depot in humans, generally considered metabolically benign or even protective relative to visceral fat [129]. However, its physiological impact is highly dependent on the anatomical site. Abdominal SAT is more closely linked to metabolic risk than gluteal-femoral SAT, which inversely correlates with T2DM [130,131]. Within abdominal SAT, a fascial division (Scarpa’s fascia) separates superficial SAT from deep SAT, the latter being histologically distinct (with irregular, flattened fat lobules) and exhibiting higher lipolysis, inflammatory gene expression (IL-6, MCP-1), and secretion of resistin with lower leptin and 11β-HSD1, paralleling visceral fat profiles [60,132]. This altered secretome may influence nearby skeletal muscle across the thin abdominal deep fascia, potentially contributing to insulin resistance [132]. Unlike the multi-layered, thicker limb fascia, which may limit such interactions, the thin, infiltrating deep fascia in the trunk facilitates direct adipose-muscle molecular crosstalk. However, the absence of such subcutaneous subdivisions in rodent models highlights the need for human-focused mechanistic studies. Overall, deep SAT correlates more strongly than superficial SAT with hepatic steatosis, inflammation, and metabolic syndrome, whereas gluteofemoral SAT often associates with metabolic protection, consistent with depot-specific lipid kinetics, adipokines, and stromal composition [133,134]. Visceral adipose tissue (VAT) expansion is more tightly linked than SAT to insulin resistance, dyslipidemia, and cardiometabolic risk, reflecting higher lipolytic flux and inflammatory tone [135].

Intermuscular adipose tissue (IMAT) accumulates within muscle fascia and, although minor in volume, strongly predicts insulin resistance and is linked to T2DM, cardiovascular disease, and sarcopenia [136,137]. Across cohorts, higher IMAT correlates with lower insulin sensitivity and poorer muscle quality in established obesity/T2DM, and human biopsy/co-culture studies show paracrine impairment of myofiber insulin action [39]. Porcine and murine studies show IMAT resembles visceral fat transcriptionally, enriching immune pathways and inflammatory microRNAs targeting IL-6/STAT3 and PPARγ-regulated genes (ADIPOQ, UCP1, FABP4), suggesting a role in promoting local inflammation and metabolic dysfunction [138,139,140]. In humans, IMAT exhibits increased expression of macrophage markers and ECM genes correlating with lower insulin sensitivity. Functionally, conditioned media from human IMAT explants elevated muscle diacylglycerol content and reduced insulin-stimulated glucose uptake to levels similar to visceral fat, supporting a direct paracrine impairment of muscle insulin signaling [39,141]. Despite these insights, comprehensive characterization of the IMAT secretome and its mechanistic impact on muscle metabolism remains limited.

Although WAT contributes less than 5% to whole-body glucose disposal, it plays a disproportionate role in systemic and local metabolic regulation by modulating non-esterified fatty acid (NEFA) and glycerol release, which impact hepatic gluconeogenesis and skeletal muscle lipid uptake [142]. In insulin-resistant adipose tissue, reductions in insulin receptor content, tyrosine kinase activity, and insulin-stimulated glucose uptake lead to impaired suppression of lipolysis, elevating circulating NEFA and exacerbating ectopic lipid accumulation in muscle [10,143]. Compartment-specific studies show higher lipolysis and reduced lipogenesis in visceral compared to subcutaneous WAT, enhancing portal lipid delivery to the liver and indirectly increasing muscle lipid overload [10]. Moreover, in obesity, WAT exhibits blunted diurnal fluctuations in net NEFA flux, limiting the dynamic storage and mobilization of triglycerides, which further promotes lipid spillover to skeletal muscle and local stromal compartments [142,143].

Expanding WAT often outpaces angiogenesis, causing hypoxia that activates HIF-1α, drives adipocyte dysfunction, and promotes extracellular matrix deposition and fibrosis [10]. Activation of mitochondrial adenine nucleotide translocase 2 (ANT2) by saturated fatty acids increases oxygen consumption, worsening hypoxia; genetic deletion of ANT2 improves oxygen balance and reduces adipose inflammation and insulin resistance despite unchanged mitochondrial mass [144]. These findings implicate fatty acid–driven mitochondrial stress as an early, therapeutically targetable driver of adipose dysfunction. Mechanical stress, hypertrophic remodeling, and nutrient overload precipitate adipocyte death and recruitment of bone marrow derived M1 macrophages, initiating a metabolic inflammatory program characterized by MCP-1, CCR2, and TNF/IL-6 signaling [145]. Importantly, local WAT inflammation can induce regional insulin resistance through cytokine release (e.g., IL-1β, TNF, IL-6) without requiring systemic cytokine elevation, underscoring a paracrine component of muscle–adipose crosstalk in obesity [146]. In insulin-resistant obese adolescents, adipose IL-6 concentrations exceed plasma by more than twentyfold, reinforcing this localized inflammatory milieu [144].

Notably, insulin resistance is increasingly recognized as arising from defects in adipocyte function and lipid buffering capacity, rather than total fat mass alone. Genome-wide association studies (GWAS) have identified loci that modulate insulin sensitivity via adipocyte differentiation and depot-specific lipid handling, reinforcing the concept that limited WAT expandability predisposes to ectopic lipid deposition in skeletal muscle and liver [147,148,149]. Epigenetic modifications in adipose tissue further influence this relationship, shaping transcriptional responses to overnutrition [150].

### 3.3. Bone Marrow Adiposity and Lipid Metabolic Dysfunction in Obesity and T2DM

Adverse lipid metabolic consequences in obesity and T2DM also extend to skeletal structures. Obesity is recognized as a major risk factor for osteoporosis [151]. This relationship is partly due to the imbalance between osteogenesis and adipogenesis, where an increase in bone marrow adipose tissue is frequently observed among individuals susceptible to osteoporosis. Increased lipid infiltration within the bone marrow, characterized by an expansion of marrow adipocytes, is commonly observed in these metabolic states and is associated with reduced bone formation and mineral density [74,80]. Notably, Dai et al. observed significantly reduced osteoblast numbers in diet-induced obese mice compared to lean mice, despite similar osteoclast percentages, suggesting enhanced bone resorption in obesity leading to osteoporosis [152]. Elevated local levels of saturated fatty acids and lipid peroxidation products stimulate osteoclastogenesis while simultaneously exerting cytotoxic and pro-oxidative effects on osteoblast precursors, impairing their differentiation and function [12]. HFD-fed mice exhibit increased serum lipids, decreased bone mineral density, and elevated circulating pro-inflammatory cytokines including IL-1 and TNF-α [153,154]. IL-1 promotes osteoclast formation by activating NF-κB and MAPK signaling via TNF receptor-associated factor 6 (TRAF6) in coordination with receptor activator of nuclear factor κB ligand (RANKL) [155]. TNF-α inhibits osteoblast differentiation and enhances osteoclast activity by activating TRAF and the NF-κB/c-Fos/NFATc1 signaling pathway independently of the RANKL/RANK system [156,157]. Additionally, HFD induces recruitment of CD11c+ macrophages secreting IL-18 and IL-1β [158].

Moreover, oxidative stress arising from excessive lipid availability exacerbates these processes by activating redox-sensitive pathways that suppress osteoblastogenesis and promote osteoclastic activity [74,80]. Obese individuals exhibit nearly twice the oxidative stress markers, such as hydrogen peroxide and malondialdehyde, compared to normal-weight counterparts [159]. Oxidized low-density lipoprotein, a prominent oxidative stress marker, significantly contributes to obesity-related bone damage [160]. Hyperlipidemia disrupts mitochondrial integrity, impairs ATP production, and reduces antioxidant enzyme activities, leading to increased ROS accumulation [161]. HFD mice display reduced total antioxidant capacity and lower superoxide dismutase levels, essential for bone strength [162]. Hyperlipidemia also suppresses Nrf2-mediated antioxidant responses in bone tissue [153]. Excessive ROS inhibits the Wnt/β-catenin pathway and decreases BMP2 and Runx2 expression, impairing osteoblast activity [163]. Oxidized lipids further promote adipogenesis through PPARγ while inhibiting β-catenin-driven osteogenesis [164]. Additionally, HFD reduces the glutathione/oxidized glutathione ratio, compromising bone formation and elevating bone resorption markers such as cross-linked N-telopeptides of type I collagen [165]. It also enhances osteoclast differentiation by suppressing the Nrf2/HO-1/catalase signaling pathway [166]. Overall, these alterations illustrate how pathological lipid dysregulation at the muscle–connective tissue interface in obesity and T2DM extends into bone, underscoring a broader network of interrelated musculoskeletal–adipose–osseous disturbances.

**Figure 3:**
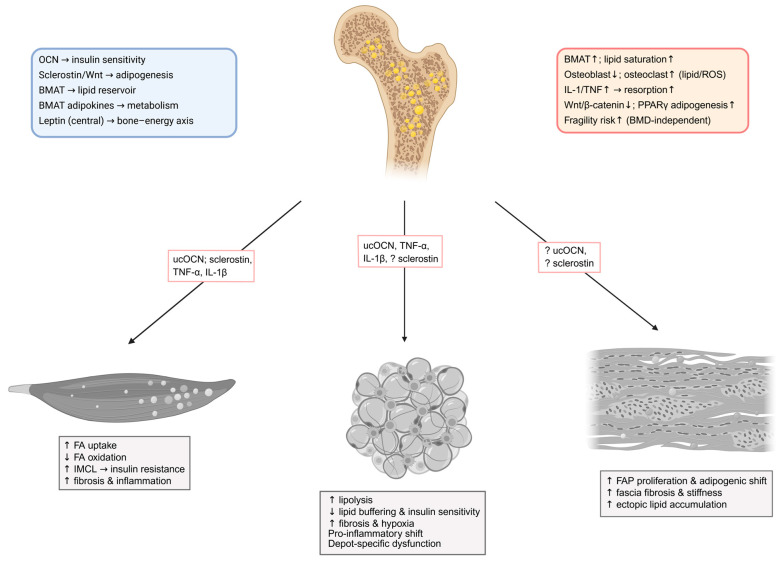
Bone tissue dysregulation and crosstalk with proximal connective tissues in obesity and type 2 diabetes. Schematic overview of physiological bone marrow regulation of metabolism (blue) contrasted with dysregulation in obesity and type 2 diabetes (orange), with representative bone/BMAT-derived mediators directed to skeletal muscle, adipose tissue, and fascia and the corresponding alterations in each target tissue. Tissue layout and lipid-relevant constituents: bone (top; bone cells and BMAT adipocytes), skeletal muscle (bottom left; metabolically distinct fibers with intracellular/extracellular lipid pools), adipose (bottom middle; white, beige, and brown adipocytes), and fascia (bottom right; collagen-rich extracellular matrix, fibroblasts and FAPs). Physiologically, osteocalcin supports insulin sensitivity, Wnt–sclerostin signaling constrains adipogenesis, BMAT acts as a lipid reservoir and source of adipokines, and central leptin links bone to energy balance. In obesity/T2DM, BMAT and lipid saturation increase; osteoblast activity declines while osteoclast activity rises with lipid/ROS stress; IL-1/TNF promote resorption; Wnt/β-catenin signaling is reduced with PPARγ-driven adipogenesis; fragility risk increases independent of BMD. Bone/BMAT-derived mediators are indicated along arrows to muscle, adipose, and fascia, and the target panels summarize downstream outcomes. Abbreviations: BMAT, bone marrow adipose tissue; BMD, bone mineral density; FA, fatty acid; FAPs, fibro-adipogenic progenitors.; IL, interleukin; IL-1β, interleukin-1 beta; IMCL, intramyocellular lipid; OCN, osteocalcin; PPARγ, peroxisome proliferator-activated receptor-gamma; ROS, reactive oxygen species; TNF-α, tumor necrosis factor-alpha; ucOCN, undercarboxylated osteocalcin; Wnt, Wnt signaling; β-catenin, beta-catenin.

In obesity and T2DM, BMAT typically expands and exhibits compositional shifts characterized by lower unsaturation and higher saturation of marrow lipids—features repeatedly linked to fragility fracture risk independent of areal bone mineral density (BMD) [167,168]. MRI-based proton density fat fraction (PDFF) and ^1^H-MRS now quantify BMAT quantity and composition, with several human studies showing higher BMAT or more saturated profiles in diabetes and metabolic syndrome, and dynamic improvement of BMAT composition paralleling glycemic benefit after bariatric surgery [169,170]. Emerging data also indicate sex-specific relationships between T2DM, circulating lipids, and BMAT composition [171]. Collectively, these findings position BMAT as a metabolically responsive depot that may contribute to skeletal fragility and altered marrow niche function in diabetes, while causality and mechanisms remain active areas of investigation. Figure 3 summarizes physiological bone–marrow regulation of metabolism and its alterations in obesity and T2DM, and illustrates bone-derived mediators that signal to skeletal muscle, adipose tissue, and fascia.

### 3.4. Fascia and Fibro-Adipogenic Remodeling in Obesity and T2DM

Emerging evidence indicates that the pathological remodeling of local connective tissues in obesity and T2DM extends to the fascia. In T2DM, ultrasound and elastography studies show that the plantar fascia and sometimes the crural fascia are thicker and display altered mechanical properties compared with healthy controls [172,173]. These changes plausibly reflect advanced glycation end-product (AGE) accumulation in long-lived fascial collagens and hyaluronan densification, both of which can alter tissue viscoelasticity and sliding between layers in diabetes and obesity [174,175]. The neurovascular organization of human fascia, including perivascular innervation and intrinsic lymphatic vessels, suggests that such matrix remodeling could influence sympathetic tone, microvascular perfusion, and immune trafficking in adjacent adipose and muscle compartments, although causal links to systemic insulin resistance require prospective human studies [88,89].

The muscular fascia harbors FAPs that under physiological conditions support extracellular matrix maintenance and aid in muscle regeneration [84,85,86,87]. However, under conditions of chronic metabolic stress, these FAPs exhibit a maladaptive shift toward excessive adipogenesis and fibrogenesis, contributing to fatty infiltration and fibrotic thickening of the fascia. Chronic low-grade inflammation biases the myofiber microenvironment toward signals that stabilize FAP survival and fibrogenic conversion. Early pro-inflammatory macrophage-derived TNF-α can trigger apoptosis of excess FAPs to limit fibrosis; with persistent metabolic stress, TGF-β and related pro-resolving signals from macrophage subsets favor myofibroblast differentiation and extracellular-matrix deposition, while also promoting fatty infiltration when adipogenic cues dominate. In mouse models, restoring TNF-sensitive apoptosis reduces fibrosis, and Hippo–YAP/TAZ nodes modulate these outcomes by integrating inflammatory and TGF-β inputs within FAPs [92,176,177]. Human single-cell datasets and ex vivo studies corroborate FAP heterogeneity and identify MME^+^ adipogenic FAPs in fatty-infiltrated human muscle, supporting a conserved mechanism that links stromal–immune crosstalk to IMAT accumulation in metabolic disease [92,178]. In injury/heterotopic ossification, fascia/muscle FAPs can undergo osteogenic conversion under BMP/Activin A–ACVR1 signaling, indicating osteogenic potential of fascia-resident cells; however, this reflects the response to BMP pathway activation, not a fascia-origin-secreted mediator affecting bone in metabolic disease [179].

Obesity increases FAPs in skeletal muscle [13,94,180], which differentiate into adipocytes and fibroblasts, driving intramyocellular lipid accumulation, fibrosis, insulin resistance, and impaired glucose uptake. In a high-fat diet mouse model, chronic obesity elevated FAP proliferation, adipocyte infiltration, and collagen deposition in the diaphragm, leading to contractile and respiratory dysfunction, with thrombospondin 1 identified as a key obesity-related stimulator of FAP expansion, highlighting FAPs as potential targets to prevent skeletal muscle remodeling and dysfunction in obesity [13,180,181]. This pathological remodeling not only alters the mechanical properties of fascia—increasing stiffness and disrupting force transmission—but also creates a microenvironment that may perpetuate local lipid dysregulation and inflammation. The resulting fibroadipogenic expansion of the fascia thus represents an additional depot of ectopic lipid accumulation directly within the musculoskeletal connective tissue interface.

**Figure 4:**
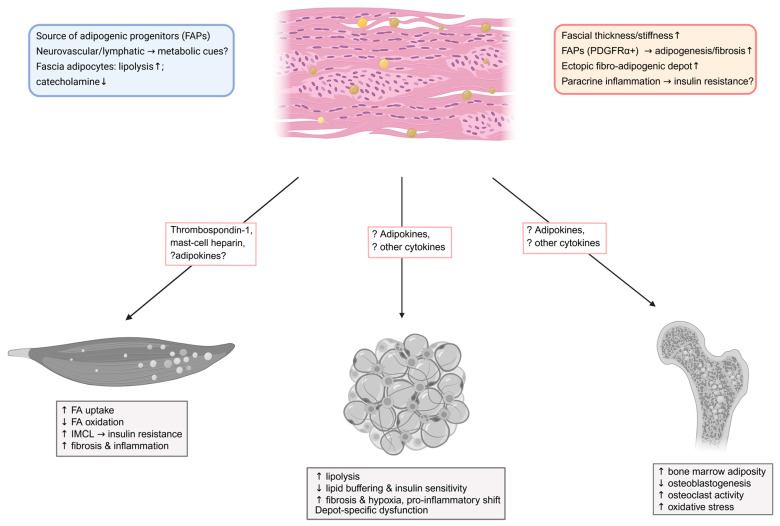
Fascia dysregulation and crosstalk with proximal connective tissues in obesity and type 2 diabetes. Schematic overview of physiological fascia features (blue) versus dysregulation in obesity/T2DM (orange), with representative fascia-derived mediators directed to skeletal muscle, adipose tissue, and bone and the corresponding alterations in each target tissue. Tissue layout and lipid-relevant constituents: fascia (top; collagen-rich extracellular matrix with FAPs (PDGFRα^+^) and fascia adipocytes, interfacing neurovascular/lymphatic networks), skeletal muscle (bottom left; metabolically distinct fibers with intra- and extracellular lipid pools), adipose (bottom middle; white, beige, and brown adipocytes), and bone (bottom right; bone cells and bone-marrow adipocytes). In physiological conditions, fascia provides a stromal niche for adipogenic progenitors and adipocytes, may relay metabolic cues via neurovascular/lymphatic interfaces, and may exhibit lipolytic activity under low catecholaminergic drive. In obesity/T2DM, fascia thickens and stiffens; FAPs (PDGFRα^+^) expand with adipogenic/fibrotic conversion, ectopic fibro-adipogenic depots increase, and paracrine inflammation may contribute to insulin resistance. Fascia-derived mediators are labeled on the arrows to muscle, adipose and bone tissues; question marks indicate hypothesized mediators or links; and target panels summarize downstream outcomes. Abbreviations: FA, fatty acid; FAPs, fibro-adipogenic progenitors; IMCL, intramyocellular lipid; PDGFRα, platelet-derived growth factor receptor-α; T2DM, type 2 diabetes mellitus.

Although, in obesity and T2DM, fascia may modulate local lipid handling at the muscle–adipose interface, direct lipid-handling functions in humans remain to be clearly demonstrated. Clinically testable questions directly relevant to lipid dysmetabolism include whether lower-limb fascial stiffness and thickness measured by shear-wave elastography associate with adjacent IMAT volume and limb-specific insulin sensitivity after accounting for SAT and VAT; whether weight loss or GLP-1RA/SGLT2 therapy reduces fascial stiffness in parallel with increased microvascular flow and improved fatty-acid oxidation; whether matrix glycation and hyaluronan remodeling correlate with impaired transcapillary fatty-acid flux and intramyocellular DAG and ceramide accumulation; and whether perivascular innervation or lymphatic features within fascia predict regional NEFA spillover and IMAT expansion [172,173,174]. FAP-centered hypotheses include whether fascial and perimysial FAPs (PDGFRα^+^) expand or adopt MME-positive adipogenic states in proportion to IMAT burden and insulin resistance, and whether macrophage-derived TNF-α versus TGF-β signaling shifts FAP fate toward apoptosis control versus fibrogenic or adipogenic remodeling [92,178]. Figure 4 summarizes physiological features of fascia tissue relevant to lipid handling and their pathological alterations in obesity and T2DM, and illustrates known and potential fascia-associated mediators communicating with skeletal muscle, adipose tissue, and bone.

## 4. Molecular Mediators and Mechanisms of Local Crosstalk Driving Lipid Dysregulation and Insulin Resistance

**Figure 5:**
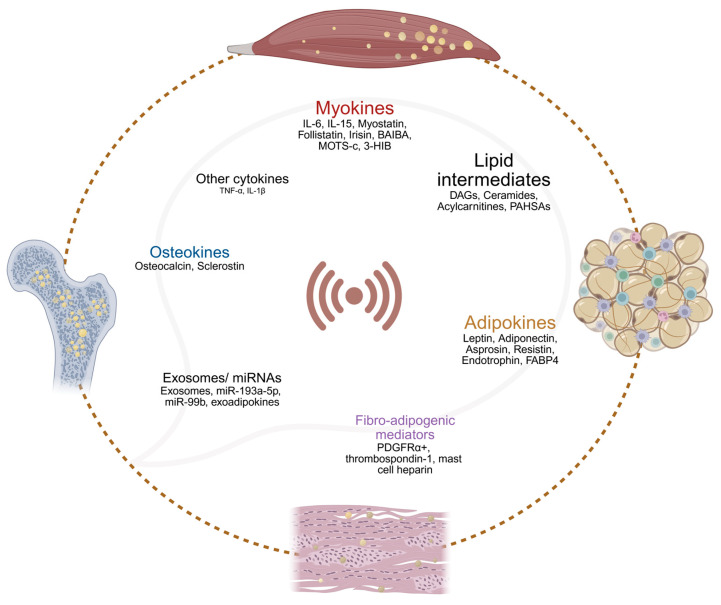
Integrative overview of inter-tissue interactions and mediator categories in lipid dysregulation in obesity and type 2 diabetes. Conceptual schematic summarizing crosstalk among skeletal muscle (top), adipose depots (right), bone (left), and fascia (bottom), and the mediator classes with representative examples. Insulin sensitivity and lipid homeostasis arise from coordinated exchanges among neighboring connective tissues, mediated by tissue-derived and systemic signals that regulate fatty-acid supply and oxidation, storage buffering, and matrix properties. Myokines are muscle-derived cytokines and hormone-like proteins that transmit the contractile and metabolic state to other tissues, broadly adjusting adipose lipolysis/thermogenesis and connective-tissue remodeling. Adipokines and stromal factors are secreted by adipocytes and their stromal–immune niche and regulate muscle insulin action, substrate use, and inflammatory tone. Osteokines and bone-derived pathways originate from bone cells and BMAT and influence systemic insulin sensitivity, marrow adipogenesis, and local lipid handling. Intermediate lipids and other signaling lipid metabolites act as inter-tissue lipid-derived messengers that modulate receptor signaling and fatty-acid trafficking, thereby shaping insulin responsiveness. Exosomes and mitochondrial-derived peptides convey regulatory RNAs and proteins/peptides between tissues to coordinate transcriptional programs and metabolic set points. Abbreviations: 3-HIB, 3-hydroxyisobutyrate; BAIBA, β-aminoisobutyric acid; DAGs, diacyl-glycerols; FABP4, fatty acid-binding protein 4; IL, interleukin; IL-1β, interleukin-1 beta; miR, microRNA gene notation; miRNA, microRNA; MOTS-c, mitochondrial open reading frame of the 12S rRNA-c; PAHSAs, palmitic acid esters of hydroxystearic acids; PDGFRα+, platelet-derived growth factor receptor-alpha positive; TNF-α, tumor necrosis factor-alpha.

Convergent human and mechanistic evidence indicate that insulin resistance is a proximal driver of lipid dysregulation in skeletal muscle and contiguous connective tissues. Local lipid dysregulation in obesity and T2DM are driven by a complex network of tissue-resident signaling molecules and lipid intermediates acting across muscle, adipose tissue, fascia, and bone. Figure 5 summarizes the conceptual framework of proximal inter-tissue crosstalk mediators in lipid metabolism among different connective tissues in obesity and T2DM. The following subsections detail these mediators and the mechanisms underlying their local interactions. An extended thematic summary of the crosstalk mediators, including their principal effects on lipid metabolism and insulin signaling are also provided in Supplementary Table S1.

### 4.1. Interleukins in Local Muscle–Adipose Crosstalk During Lipid Dysregulation

During contraction and metabolic stress, skeletal muscle secretes an array of myokines that influence local adipose tissue lipid metabolism, thermogenesis, and inflammatory tone. Interleukins such as IL-6 and IL-15 represent important modulators of local interactions between skeletal muscle and adipose tissue in obesity and T2DM. IL-6 is robustly induced by muscle activity, rising up to 100-fold in circulation, and acts within muscle to enhance AMPK-mediated fatty acid oxidation [182,183,184]. Notably, IL-6 also modulates adipose tissue, with exogenous administration increasing UCP1 expression in subcutaneous WAT, suggesting a role in promoting local browning and lipid mobilization [143,185]. Under basal conditions in obesity, IL-6 is primarily derived from adipose tissue, where it contributes to a chronic pro-inflammatory state linked to impaired insulin signaling [186]. However, transient increases in muscle IL-6—induced by contraction or exogenous infusion—have been shown to acutely enhance insulin-stimulated glucose uptake and lipid oxidation in skeletal muscle, suggesting context-dependent effects [187,188]. In individuals with T2DM, IL-6 infusion can also improve glucose tolerance, potentially via augmented GLP-1-mediated insulin secretion or delayed gastric emptying, though these effects do not necessarily indicate direct muscle–adipose metabolic crosstalk [189].

IL-15, originally identified as an adipokine but also secreted by muscle and immune cells, regulates adiposity by enhancing lipolysis and reducing lipid synthesis via JAK/PKA pathways, supporting a metabolic milieu that favors lipid utilization over storage [190,191]. IL-15 exhibits negative correlations with fat mass and may support local metabolic interactions by promoting PPAR-driven fatty acid oxidation and shifting the cytokine milieu toward a less inflammatory state [190,191]. However, skeletal muscle expression of IL-15 does not consistently differ between lean and obese individuals, and adipose tissue remains a likely dominant source [190]. Notably, controlled exercise studies in people with obesity and T2DM have revealed no significant alterations in myocellular expression or systemic levels of key myokines (including IL-6, IL-15, FGF21, and angiopoietin-like 4) compared to lean controls, challenging the notion that dysregulation of these myokines underpins insulin resistance in human skeletal muscle [192,193]. These findings underscore the need for more targeted investigations into how local interleukin dynamics specifically shape lipid handling and insulin sensitivity within skeletal muscle–adipose–stromal microenvironments.

### 4.2. Other Myokines and Muscle-Derived Factors in Local Lipid Dysregulation

Beyond classical myokines, skeletal muscle secretes a variety of cytokines, chemokines, and metabolites that may indirectly influence local lipid handling and insulin sensitivity within muscle–adipose–connective tissue microenvironments in obesity and T2DM. Myostatin, beyond its canonical inhibition of muscle growth, directly regulates adipose mass and lipid turnover. Elevated in obesity, myostatin promotes WAT expansion and insulin resistance, whereas its genetic or pharmacologic inhibition reduces fat mass and enhances insulin sensitivity [194]. Follistatin, which antagonizes myostatin, rises with exercise and may limit WAT growth, reinforcing muscle’s paracrine control over local lipid handling [195,196,197]. Exercise-induced activation of PPARγ-coactivator 1α (PGC1α) in muscle promotes the release of several factors with potential local metabolic effects. Irisin, derived from cleavage of FNDC5, has been linked to the beiging of WAT in rodents; however, human data on circulating irisin levels remain highly variable, casting doubt on its relevance to local lipid partitioning in skeletal muscle or connective tissues in obesity and T2DM [198,199,200]. By contrast, PGC1α also regulates secretion of amino acid metabolites such as β-aminoisobutyric acid (BAIBA) and 3-hydroxyisobutyrate (3-HIB). While BAIBA enhances fatty acid oxidation and reduces hepatic gluconeogenesis, circulating levels are paradoxically higher in T2DM and inversely associated with insulin secretion [201]. Meanwhile, 3-HIB, a valine metabolite, has been shown to promote transendothelial fatty acid transport, increasing intramyocellular diacylglycerol accumulation and triggering PKCθ-mediated insulin resistance in skeletal muscle [202]. Emerging data also point to skeletal muscle–derived mitochondrial signals. The mitochondrial-encoded peptide MOTS-c is inversely correlated with glycaemia and HbA1c in T2DM and may protect against oxidative stress and myostatin-driven muscle loss [203,204]. Together, these findings underscore a growing repertoire of muscle-derived factors that could contribute to local tissue lipid dysregulation and insulin resistance, warranting more targeted investigation in the context of obesity and T2DM.

### 4.3. Leptin and Adiponectin in Local Lipid Dysregulation and Adipose–Muscle Crosstalk

Adipose tissue secretes key peptide hormones, known as adipokines, that act locally to modulate the function of adjacent tissues. Among adipose-derived hormones, leptin and adiponectin are key modulators of local lipid handling and insulin responsiveness in skeletal muscle and adipose tissue. While best known for its central effects on appetite, leptin also has significant local actions within the connective tissue microenvironment [205]. Leptin, predominantly secreted by WAT, is a pleiotropic hormone that regulates energy intake, expenditure, and lipid metabolism by activating leptin receptor (LepR)–STAT3 signaling in the hypothalamus and peripheral tissues [206,207]. Experimental models show that leptin can directly reduce intramyocellular lipid content by promoting fatty acid oxidation and limiting lipid storage, even independently of its central appetite-regulating effects [208,209]. However, obesity commonly leads to leptin resistance, characterized by diminished cellular responsiveness despite elevated circulating levels. In muscle, this impairs lipid oxidation and fosters intramyocellular lipid accumulation, contributing to local insulin resistance [210,211]. Parallel effects in adipose depots and vascular tissues exacerbate inflammation and fibrosis, reinforcing regional metabolic dysfunction [206,207,210]. However, human studies indicate that variability in tissue leptin sensitivity contributes to inconsistent associations between circulating leptin and insulin resistance across individuals [212].

Adiponectin, predominantly secreted by adipocytes, exerts potent anti-inflammatory, insulin-sensitizing, and lipid-modulating actions. It enhances skeletal muscle lipid oxidation via activation of the AMPK and PPAR pathways, while concurrently suppressing hepatic gluconeogenesis [213,214]. It also promotes ceramidase activity via its receptors AdipoR1 and AdipoR2, lowering tissue ceramide levels and protecting insulin signaling [215]. In obesity, both circulating adiponectin and receptor responsiveness are reduced, impairing muscle fatty acid oxidation and exacerbating lipid deposition and insulin resistance [214]. Locally, low adiponectin levels are linked to increased inflammatory signaling and altered extracellular matrix remodeling in adipose and muscle tissues, potentially worsening lipotoxicity and fibrosis [214,216].

### 4.4. Other Adipokines Involved in Local Lipid Metabolism and Insulin Sensitivity

Beyond leptin and adiponectin, several other adipokines secreted from dysfunctional adipose tissue modulate local metabolic processes within skeletal muscle and proximal connective tissues, with varying implications for lipid handling and insulin sensitivity. Unlike rodents, where resistin is primarily adipocyte-derived, in humans it is mainly secreted by macrophages within adipose tissue. Resistin links local inflammation to metabolic impairment by activating NF-κB and MAPK pathways, thereby exacerbating insulin resistance and promoting lipid accumulation in nearby skeletal muscle and liver [217]. In perivascular and adipose environments, resistin-induced inflammation contributes to fibrotic remodeling and endothelial dysfunction, reinforcing the connection between regional adipose dysfunction, muscle lipid overload, and cardiovascular risk in obesity and T2DM [217,218]. FGF21, predominantly released by BAT, induces mitochondrial remodeling in skeletal muscle, enhances oxidative capacity, and shifts myofiber phenotype via p38 MAPK and suppression of TGF-β1 signaling, underscoring integrated muscle–BAT metabolic regulation [215]. Fatty acid-binding protein 4 (FABP4), abundantly produced by adipocytes, is released under lipolytic conditions and has been shown to enhance hepatic gluconeogenesis; in obesity and T2DM, circulating FABP4 levels are elevated, yet Mendelian randomization suggests no direct causal impact on insulin resistance [216,219,220]. However, higher FABP4 concentrations have been associated with increased cardiovascular mortality in individuals with diabetes, hinting at its broader metabolic relevance [221]. Retinol-binding protein 4 (RBP4), although initially characterized as a hepatokine, is also secreted by adipocytes and can activate local immune cells, fostering low-grade inflammation. Its relationship with insulin resistance and cardiovascular risk appears complex, with recent studies highlighting sex-specific and U-shaped associations in T2DM [222,223]. Another notable adipokine is endotrophin, a cleavage product of collagen VI α3, which drives adipose tissue fibrosis, inflammation, dyslipidemia, and insulin resistance in experimental models [224,225]. In people with T2DM, elevated circulating endotrophin predicts a poorer response to insulin-sensitizing treatments and correlates with increased cardiovascular and renal risk [226,227]. Additionally, adipokines such as asprosin—which rises early in T2DM and promotes insulin resistance in skeletal muscle—illustrate the dual roles many of these factors play, as asprosin may simultaneously exert cardioprotective and insulinotropic effects [228,229]. Conversely, adipsin, generally reduced in T2DM, has been implicated in modulating insulin secretion, although its links to impaired glucose or lipid metabolism in humans remain inconclusive [230]. Collectively, these findings underscore the complexity of adipose-derived signals and their variable impact on local metabolic crosstalk, influencing lipid partitioning and insulin responsiveness within skeletal muscle and nearby connective tissues in obesity and T2DM.

### 4.5. Lipid Intermediates and Signaling Lipids Shaping Local Metabolic Communication

In obesity and T2DM, the local accumulation of bioactive metabolites—including diacylglycerols, ceramides, and branched-chain amino acid (BCAA) catabolites—within skeletal muscle, adipose tissue, bone, and fascia contributes directly to tissue-specific insulin resistance and metabolic dysfunction. Elevated intracellular accumulation of sn-1,2-diacylglycerols (DAGs) within skeletal muscle and adipose tissue has been causally linked to impaired insulin signaling. These DAGs arise primarily from re-esterification of excess fatty acids and become enriched in the plasma membrane of myocytes and adipocytes, where they activate novel protein kinase C (nPKC) isoforms, including PKCθ, PKCε, and PKCδ [16,231]. Activated nPKCs phosphorylate critical components of the insulin signaling cascade, including serine 1101 of insulin receptor substrate 1 (IRS1), thereby reducing phosphatidylinositol-3-kinase (PI3K) activity and downstream glucose uptake [16]. This mechanism has been confirmed in human studies using intravenous lipid infusions to raise NEFA levels, which led to DAG accumulation in skeletal muscle membranes and correlated with reduced glycogen synthesis and insulin-stimulated glucose disposal [232]. Importantly, in the absence of systemic inflammatory or oxidative stress markers, these studies suggest that DAG–nPKC signaling plays a primary role in initiating insulin resistance at the tissue level [16,231]. Elevated levels of C18-containing DAG species and activated PKCθ are consistently observed in skeletal muscle of individuals with obesity or T2DM and correlate strongly with metabolic impairment [231]. Similar DAG-dependent mechanisms operate in adipose tissue, where adipose-specific PKCε knockdown in mice improves both local insulin sensitivity and whole-body glucose homeostasis [16].

Ceramides, bioactive sphingolipids synthesized de novo from saturated fatty acids such as palmitate, accumulate in skeletal muscle and adipose tissue under conditions of lipid excess, such as in obesity and T2DM. Their local production is enhanced by increased fatty acid influx, and while some may be transported via lipoproteins or extracellular vesicles, tissue-specific origins and targets remain incompletely defined [233,234]. Within insulin-sensitive tissues, ceramides disrupt distal insulin signaling by inhibiting AKT (protein kinase B) activation. Mechanistically, ceramides either activate protein phosphatase 2A (PP2A), which dephosphorylates AKT at Thr308/Ser473, or induce atypical protein kinase C (aPKCλ/ζ) activity, which interferes with AKT’s membrane translocation and binding to phosphatidylinositol (3,4,5)-trisphosphate (PIP3) [235,236]. In both skeletal muscle and adipose tissue, this leads to impaired glucose uptake and anabolic signaling. Elevated levels of C16:0 and C18:0 ceramides in muscle and adipose depots have been directly associated with insulin resistance in human studies [237,238].

In obesity and T2DM, saturated fatty acids such as palmitate contribute to insulin resistance in skeletal muscle and adipose tissue not only through ceramide synthesis but also by promoting endoplasmic reticulum (ER) stress. Palmitate-derived sphingosine-1-phosphate has been shown to attenuate AKT phosphorylation in hepatocytes, and similar mechanisms may be relevant in muscle and adipose cells, though direct evidence remains limited [239,240]. ER stress in metabolic tissues is driven by lipid overload, with key effectors including dihydroceramides, c-Jun N-terminal kinase (JNK), and nuclear factor-κB (NF-κB), all of which impair insulin signaling through inflammatory or stress-response pathways [111]. In adipose tissue, palmitate-induced ER stress activates macrophages, triggering the release of cytokines such as TNF-α and IL-6, which contribute to local paracrine inhibition of insulin action [241,242]. However, translation of these findings to humans has yielded variable outcomes. This variability likely reflects tissue specificity, lipid species diversity (e.g., chain length, saturation), and intracellular compartmentalization (membrane-bound vs. cytosolic pools), all of which influence whether lipid molecules act as bioactive signals or simply structural components. Moreover, certain sphingolipids may play dual roles within pro- and anti-inflammatory networks, complicating their classification as uniformly pathogenic [243,244].

Pro-inflammatory lipids, including lysophosphatidic acid, oxylipins, and oxygenated polyunsaturated fatty acids, have been shown to activate local immune pathways and impair insulin sensitivity in adipose and muscle tissues [59,245]. Conversely, several lipids exhibit anti-inflammatory and insulin-sensitizing effects, such as palmitoleic acid, cis-7-hexadecenoic acid, ω-3 polyunsaturated fatty acids, and palmitic acid esters of hydroxystearic acid (PAHSAs) [246,247]. In adipose tissue, PAHSAs enhance glucose uptake via GLUT4 translocation, reduce macrophage activation, and stimulate GLP-1 and insulin secretion through G protein-coupled receptors GPCR120 and GPCR40, respectively [246]. These effects represent mechanisms of local metabolic crosstalk, potentially beneficial in counteracting lipid-induced insulin resistance. Additionally, accumulation of long-chain acylcarnitines, resulting from incomplete fatty acid oxidation, is observed in insulin-resistant skeletal muscle and correlates with mitochondrial stress [248]. Short-chain acylcarnitines derived from branched-chain amino acid metabolism are similarly associated with metabolic inflexibility in T2DM, though their precise role in local tissue signaling remains unclear [249].

### 4.6. miRNAs and Exosomes in Local Adipose–Muscle Crosstalk

Intercellular communication is further refined through extracellular vesicles such as exosomes, which ferry microRNAs (miRNAs), lipids, and proteins across tissues, thereby orchestrating local and systemic metabolic regulation [250,251]. Skeletal muscle-derived exosomes, particularly those enriched in specific miRNAs, can modulate adipocyte differentiation, lipolysis, and insulin sensitivity [252]. Adipose-derived microRNAs (miRNAs) packaged in exosomes are emerging as important modulators of local tissue interactions. Specific miRNAs, such as miR-193a-5p, have been linked to altered glucose metabolism and increased risk of T2DM in large population studies, while miR-99b appears to influence lipid handling by regulating hepatic FGF21, with potential local analogs in skeletal muscle and adipose cross-communication [253,254,255,256,257]. Exosomes from stressed adipocytes also carry diverse cargos—including oxidatively modified mitochondrial fragments and protein factors termed exoadipokines—that can influence metabolism in recipient cells. In murine models, adipocyte-derived exosomes transfer damaged mitochondria to cardiac tissue, altering oxidative stress responses; though similar mechanisms may exist locally between adipose depots and muscle, their direct contribution to perimuscular lipid dysregulation and insulin resistance remains underexplored [257,258,259]. Exosomes isolated from human adipocytes have been shown to contain a broad array of signaling proteins, reinforcing their potential role as vehicles of local paracrine communication in metabolic disease [257].

### 4.7. Compartment-Specific Adipose Secretomes Influence Local Muscle and Stromal Metabolism

The anatomical localization of adipose depots critically shapes their secretory profiles, thereby modulating local metabolic crosstalk with skeletal muscle and adjacent connective tissues in obesity and T2DM. Distinct fat compartments—such as subcutaneous (SAT), visceral (VAT), intermuscular, and perivascular adipose tissue—exhibit unique secretomes that differentially affect nearby metabolic and structural cells [11,136,260]. Notably, VAT secretes higher levels of pro-inflammatory cytokines including IL-6, TNF-α, IL-8, and IL-12p70, as well as matrix metalloproteinases and plasminogen activator inhibitor-1 (PAI-1), compared to SAT [10,11,136,260,261]. Conditioned media from VAT elicits stronger lipotoxic and inflammatory responses in cultured myotubes, suggesting a direct mechanism by which visceral fat exacerbates skeletal muscle insulin resistance and lipid dysregulation [260]. These compartment-specific secretory patterns highlight how adipose depot heterogeneity can shape local lipid partitioning, extracellular matrix remodeling, and insulin sensitivity in skeletal muscle and surrounding stromal environments.

### 4.8. Roles of Myokines, Osteokines, and Adipokines in Muscle–Bone–Adipose Metabolic Interactions

Emerging data indicate that myokines such as IL-6, IL-7, IL-15, and myostatin critically influence bone remodeling, thereby indirectly shaping local lipid handling within skeletal and bone compartments. Exercise-stimulated muscle-derived IL-6, while broadly anti-inflammatory and improving glucose metabolism [262], paradoxically promotes osteoclastogenesis by increasing RANK/RANKL signaling and enhancing bone resorption [263], processes that can alter lipid substrate demand during bone turnover. Similarly, IL-7 and IL-15 released from skeletal muscle stimulate osteoclast differentiation via RANKL catabolism [264,265], further driving bone catabolism and potentially influencing regional energy flux. Myostatin, a TGFβ family cytokine upregulated in sarcopenia and muscle disuse, not only impairs muscle lipid oxidative capacity but also induces osteoclastogenesis and suppresses osteoblast function; conversely, its inhibition (via follistatin or ultrasound therapy) enhances bone formation [264,266,267]. Mechanistically, myostatin downregulates osteocyte exosomal miR-218, increasing sclerostin, RANKL, and DKK1 expression and suppressing Wnt-driven osteogenesis [267], indirectly modifying the lipid microenvironment within bone. In contrast, muscle-expressed IGF-1 and fibroblast growth factor 2 (FGF2) support osteoblast proliferation and survival [268,269], potentially sustaining bone lipid metabolism under anabolic conditions. While FGF21 is linked to enhanced glucose uptake, it may also contribute to bone loss, though in vivo data remain inconsistent [270,271]. Irisin, cleaved from FNDC5, improves muscle oxidative metabolism and enhances osteoblastogenesis and bone mass, likely via MAPK activation [272], integrating skeletal muscle energy dynamics with bone lipid remodeling.

Osteocalcin (OCN), secreted by osteoblasts, has emerged as a critical bone-derived regulator of muscle lipid metabolism. Undercarboxylated osteocalcin (ucOCN) enhances insulin sensitivity, glucose uptake, and muscle mass through GPRC6A-dependent pathways [75], indirectly favoring lipid oxidation by improving substrate switching. This effect depends on muscle IL-6, establishing a bidirectional IL-6/OCN feedback loop essential for exercise-mediated metabolic adaptation [273]. Serum sclerostin (Sost), an osteocyte-derived inhibitor of Wnt signaling, inversely correlates with muscle mass in sarcopenic individuals [274,275], suggesting it may suppress muscle anabolic capacity and indirectly influence intramyocellular lipid dynamics, although detailed mechanistic insights are still needed. Additionally, osteocyte sclerostin can promote adipogenesis/beiging in adipose tissue, suggesting theoretical relevance to mesenchymal stromal cells, but fascia-specific effects have not been shown [77].

Furthermore, recent findings underscore how adipokines such as leptin, adiponectin, resistin, and visfatin orchestrate lipid handling across muscle and bone. In obesity, hyperleptinemia drives IL-6 and TNF-α while reducing adiponectin, fostering insulin resistance and constraining muscle fatty acid oxidation [276]. This lipid inflexibility at the muscle level is compounded by chronic low-grade inflammation, which also increases bone marrow adiposity and accelerates osteoclastic resorption [277], linking adipose dysfunction to both muscle and bone lipid dysmetabolism. In contrast, adiponectin directly enhances skeletal muscle fatty acid oxidation and mitochondrial biogenesis via AMPK–PGC1α pathways and decreases hepatic gluconeogenesis [278], mitigating lipid spillover to skeletal and bone tissues. Exercise alleviates these adverse effects by lowering leptin, raising adiponectin, and improving systemic inflammation and insulin sensitivity [279,280], collectively counteracting local lipid overload in muscle and protecting bone integrity.

## 5. Conclusions and Future Directions

The evidence synthesized in this review establishes that the pathogenesis of lipid dysregulation and insulin resistance in obesity and T2DM is not merely a consequence of systemic factors or cell-autonomous defects, but is profoundly influenced by a complex and localized crosstalk between skeletal muscle, adipose tissue, bone, and fascia. We have moved beyond the traditional view of these as isolated entities to highlight them as an interconnected functional unit where pathological changes in one tissue propagate dysfunction in its neighbors. The central theme emerging is a vicious cycle: lipid overload initiates lipotoxicity and inflammation within each compartment, which in turn triggers the release of maladaptive signals— myokines, adipokines, osteokines, exosomal cargo, and bioactive lipid intermediates—that amplify metabolic stress across the entire microenvironment, and reinforce systemic metabolic dysfunction. This inter-tissue communication underscores why focusing solely on isolated organs may be insufficient to fully unravel the complex pathogenesis of insulin resistance and its complications.

Despite the extensive nature of the literature surveyed and our careful synthesis of available evidence, the inherent limitations of a narrative (non-systematic) review must be acknowledged. The search and selection were not designed to be exhaustive, which introduces potential selection and reporting bias, and restriction to peer-reviewed, English-language publications may exclude relevant non-English or preprint work. Moreover, mechanistic synthesis necessarily integrates results across model systems and analytical platforms; differences in experimental context, assay sensitivity, and endpoint definitions can constrain direct comparability. Several mechanistic domains therefore still rely on preclinical models and heterogeneous human methodologies (e.g., imaging/biopsy protocols, depot definitions, and BMAT quantification), which limits cross-study comparability and precludes causal inference or quantitative synthesis.

Accordingly, while substantial advances have been made in understanding inter-tissue communication in lipid metabolism, important gaps remain. The spatial and temporal behavior of lipid intermediates across muscle, adipose, bone marrow, and fascia is still incompletely mapped. The molecular circuits that connect fibro-adipogenic remodeling to myocellular insulin action are also not fully defined. Depot and lineage heterogeneity in SAT, IMAT, BMAT, and fascia-resident progenitors needs deeper resolution with single-cell and spatial multi-omics. In humans, the secretomes of IMAT and fascia-associated fat and their direct paracrine effects on muscle insulin signaling are poorly characterized. Many myokine and adipokine pathways are well described in rodents, but their causal roles in humans remain uncertain and require targeted validation. Filling these gaps is essential for identifying tissue- and compartment-specific therapeutic targets. Research priorities may include harmonized phenotyping that links imaging of ectopic depots, with paired metabolic testing and protocolized tissue sampling. We also recommend single-nucleus assays on frozen human muscle to reduce dissociation artifacts and to resolve stromal subsets, including FAPs [281]. Pilot spatial transcriptomics at the muscle–fascia–IMAT interface, combined with targeted spatial lipidomics using mass spectrometry imaging for DAGs, ceramides, and triglyceride species, can assign lipid intermediates to cellular neighborhoods in situ [282,283].

Future research should prioritize elucidating these local communication networks with greater precision. The application of spatially resolved omics technologies—such as spatial transcriptomics, proteomics, and lipidomics—is essential to map the molecular dialogue at the cellular interface of these tissues in situ. Coupling these datasets with quantitative measures of extracellular matrix composition and mechanics will clarify how matrix remodeling shapes metabolic crosstalk. Integrative spatial atlases that overlay human cell states (myonuclei, satellite cells, endothelial and immune cells, and defined FAP subtypes) with spatial distributions of lipid species and capillary or perivascular architecture can delineate pathways of lipid dysmetabolism. Analyses of cohorts receiving GLP-1 receptor agonists, SGLT2 inhibitors, or structured training, with paired pre- and post-intervention biopsies, can identify reversible molecular programs in obesity and T2DM. Study designs that incorporate rigorous co-registration across modalities, standardized pre-analytics, and sufficient power for multilayer endpoints will improve reproducibility and enable meaningful synthesis across studies [284,285].

Moreover, translating findings from animal models to human physiology through targeted mechanistic studies is imperative. Targeted human studies are needed to test causality and effect sizes. Over the longer term, a prospective human connective tissue resource that integrates imaging, deep metabolic phenotyping, and banked tissue under standardized pre-analytics, and that enrolls other insulin resistant phenotypes, would enable validation of mechanistic signatures across populations. Open data and harmonized ontologies will be critical to reduce batch effects and accelerate replication. Key technical hurdles to address across this roadmap include pre-analytical variability, dissociation bias in multinucleated fibers, region-of-interest selection across connective tissue planes, cross-platform normalization in spatial assays, and longitudinal retention in interventional cohorts [286]. Careful consideration of these constraints at the design and analysis stages should improve the yield and translational value of human studies in lipid dysmetabolism. Ultimately, a deeper understanding of this intricate crosstalk holds the promise of identifying novel therapeutic targets aimed not at a single tissue, but at the pathological communication network itself, offering new strategies to disrupt the cycle of lipid dysregulation and restore metabolic homeostasis.

## Data Availability

No new data were generated or analyzed in this study. All data discussed are derived from previously published articles, which are cited appropriately throughout the manuscript.

## Supplementary Materials

The following supporting information can be downloaded at: https://www.mdpi.com/article/10.3390/metabo15090581/s1, Table S1: Local crosstalk mediators regulating lipid metabolism across skeletal muscle and connective tissues in obesity and T2DM.

## Abbreviations

The following abbreviations are used in this manuscript:

3-HIB	3-hydroxyisobutyrate
AMPK	AMP-activated protein kinase
ATGL	adipose triglyceride lipase
BAIBA	β-aminoisobutyric acid
BAT	brown adipose tissue
BCAA	branched-chain amino acid
DAGs	diacylglycerols
ER	endoplasmic reticulum
FA	fatty acid
FABP4	Fatty acid-binding protein 4
FAPs	fibro-adipogenic progenitors
FATP4	Fatty Acid Transport Protein 4
FFA	free fatty acid
FGF2	fibroblast growth factor 2
FGF21	fibroblast growth factor 21
GWAS	Genome-wide association studies
HFD	high-fat diet
HSL	hormone-sensitive lipase
IGF-1	insulin-like growth factor 1
IL-1	interleukin-1
IL-6	interleukin-6
IMAT	Intermuscular adipose tissue
IMCL	intramyocellular lipids
IRS-1	insulin receptor substrate 1
LCFA	long-chain fatty acid
LDs	lipid droplets
NEFA	non-esterified fatty acid
nPKC	novel protein kinase C
OCN	Osteocalcin
PAHSAs	palmitic acid esters of hydroxystearic acid
PGC1α	PPARγ-coactivator 1α
PLIN5	perilipin 5
PPAR	peroxisome proliferator-activated receptor
RANKL	receptor activator of nuclear factor κB ligand
RBP4	Retinol-binding protein 4
ROS	reactive oxygen species
SAT	subcutaneous adipose tissue
Sost	sclerostin
T2DM	type 2 diabetes mellitus
TAG	triacylglycerol
TNF-α	tumor necrosis factor-alpha
TRAF6	TNF receptor-associated factor 6
UCP1	uncoupling protein 1
ucOCN	undercarboxylated osteocalcin
VAT	visceral adipose tissue
WAT	white adipose tissue
