# Supplementary material for: Crosstalk Between Skeletal Muscle and Proximal Connective Tissues in Lipid Dysregulation in Obesity and Type 2 Diabetes
Source: Metabolites. 2025 Aug 30;15(9):581. doi: 10.3390/metabo15090581 (PMC12471726; doi:10.3390/metabo15090581)
Supplement: Supplementary file 1 [file metabolites-15-00581-s001.zip › metabolites-3780712-supplementary.pdf]

metabolites

MDPI

Review

Crosstalk Between Skeletal Muscle and Proximal Connective Tissues in Lipid Dysregulation in Obesity and Type 2 Diabetes

Nataša Pollak [1], Efua Gyakye Janežič [1], Žiga Šink [1], Chiedozie Kenneth Ugwoke [1,*]

[1] Institute of Anatomy, Faculty of Medicine, University of Ljubljana, 1000 Ljubljana, Slovenia; Nataša Pollak, natasa.pollak@mf.uni-lj.si (ORCID: 0000-0003-3429-4441); Efua Gyakye Janežič, efua.ewusi-brown@mf.uni-lj.si (ORCID: 0009-0000-4414-6667); Žiga Šink, ziga.sink@mf.uni-lj.si (ORCID: 0009-0007-2454-6592); Chiedozie Kenneth Ugwoke, chiedozie.ugwoke@mf.uni-lj.si (ORCID: 0000-0003-4143-369X)

* Correspondence: chiedozie.ugwoke@mf.uni-lj.si

Table S1. Local crosstalk mediators regulating lipid metabolism across skeletal muscle and connective tissues in obesity and T2DM.

Category	Key molecules	Target Tissues	Main Effects in Obesity / T2DM State
Myokine	IL-6	Muscle, adipose, bone	↑ FAO via AMPK/PGC-1α in muscle, ↑ lipolysis and browning (UCP1) in WAT, ↑ bone lipid turnover via RANKL, paracrine muscle–adipose signaling, chronic IL-6 ↑ in adipose → inflammation, ↓ insulin sensitivity, local WAT insulin resistance, ↑ bone resorption.
	IL-15	Adipose, bone	↑ Lipolysis and FAO via AMPK/PGC-1α, activates PPARδ, ↓ lipogenesis via JAK/PKA, ↑ bone resorption via RANKL; IL-15 mainly adipose-derived, unchanged in muscle, unclear impact on insulin resistance, may preserve FAO and anti-inflammatory tone.
	Myostatin	Adipose, bone	↓ FAO via suppressed oxidative pathways, ↑ WAT mass, ↑ fibrosis, ↑ osteoclastogenesis, ↓ osteoblast function, alters bone lipid turnover; ↑ myostatin in obesity promotes WAT expansion, sarcopenia, insulin resistance, and impaired lipid handling in muscle and bone.
	Follistatin	Muscle, adipose, bone	Antagonizes myostatin → ↓ WAT expansion, ↑ muscle FAO, ↑ bone formation; ↑ follistatin with exercise, may limit adipose growth and support bone/muscle health, not consistently altered in obesity/T2DM.
	Irisin	Adipose, bone	↑ FAO and mitochondrial gene expression in muscle, ↑ beiging in adipose, ↑ osteoblast activity; circulating levels variable in humans, unclear role in lipid metabolism during obesity/T2DM.
	BAIBA	Muscle	↑ FAO and mitochondrial activity in muscle, ↓ hepatic gluconeogenesis; paradoxically ↑ circulating BAIBA in obesity/T2DM, inversely linked to insulin secretion.
	MOTS-c	Muscle	↓ Oxidative stress, ↑ insulin sensitivity, ↓ myostatin-driven lipid dysregulation in muscle; ↓ in T2DM, linked to poor glycemic control and ↑ muscle lipid accumulation.
	3-HIB	Muscle	↑ Trans-endothelial FA transport, ↑ DAG in muscle, driven by PGC1α and valine catabolism; ↑ in T2DM, promotes lipid overload and PKCθ-mediated insulin resistance.

Adipokine	Leptin	Muscle, bone	↑ FAO in muscle, ↓ IMCL, modulates lipid metabolism via LepR–STAT3 and inflammation; in obesity/T2DM: ↑ leptin → resistance, ↓ FAO, ↑ IMCL, ↑ IL-6/TNFα, ↑ bone marrow adiposity.
	Adiponectin	Muscle, bone	↑ FAO via AMPK/PPAR, ↑ mitochondrial biogenesis, ↑ ceramidase activity → ↓ ceramides, anti-inflammatory: ↓ adiponectin & receptor response, ↓ FAO, ↑ lipid accumulation & insulin resistance, ↑ local inflammation & fibrosis, contributes to muscle/bone lipid dysregulation.
	Asprosin	Muscle	↑ Glucose release, may support insulin secretion, pleiotropic; in obesity/T2DM: ↑ early in T2DM, promotes insulin resistance in muscle, linked to adverse metabolic profile.
	Resistin	Muscle	Activates NF-κB/MAPK, ↑ lipid accumulation in muscle, links inflammation to lipid dysregulation; in obesity/T2DM: ↑ in macrophage-rich adipose, ↑ insulin resistance, fibrosis, endothelial dysfunction, contributes to lipid overload and CV risk.
	Endotrophin	Muscle, adipose	↑ Fibrosis, inflammation, dyslipidemia, insulin resistance in adipose; in obesity/T2DM: ↑ endotrophin, predicts poor response to insulin-sensitizers.
	FABP4	Muscle	↑ with adipocyte lipolysis, modulates PPARγ-linked lipid metabolism; in obesity/T2DM: ↑ FABP4, linked to inflammation and metabolic dysfunction.
Osteokines	Osteocalcin (ucOCN)	Muscle	↑ Insulin sensitivity, glucose uptake, lipid oxidation via GPRC6A and IL-6–ucOCN loop; in obesity/T2DM: ↓ ucOCN, → impaired insulin sensitivity, ↓ lipid oxidation, disrupted IL-6–OCN signaling.
	Sclerostin (SOST)	Muscle	↓ Wnt signaling → ↓ muscle anabolic tone, ↑ adipogenesis, indirect ↑ lipid accumulation; in obesity/T2DM: ↑ SOST, possibly promotes IMCL accumulation via ↓ muscle mass.
Lipid Intermediates & Signaling Lipids	DAGs	Muscle, adipose	↑ DAG → activates PKCθ/ε/δ → ↓ IRS1–PI3K–AKT → ↓ glucose uptake, lipotoxicity in muscle/adipose; in obesity/T2DM: ↑ DAGs → nPKC activation → insulin resistance, strong link to metabolic dysfunction.
	Ceramides	Muscle, adipose	↑ Ceramides → PP2A/aPKCλ/ζ activation → ↓ AKT → ↓ glucose uptake, ↑ ER stress & ROS, lipotoxicity; in obesity/T2DM: ↑ ceramides in muscle/adipose, ↓ adiponectin → ↓ ceramidase → accumulation, insulin resistance.
	Acylcarnitines	Muscle	↑ Acylcarnitines = incomplete β-oxidation, signal mitochondrial stress, ↓ metabolic flexibility; in obesity/T2DM: ↑ long-/short-chain acylcarnitines in insulin-resistant muscle → mitochondrial dysfunction, metabolic inflexibility.
	PAHSAs	Adipose, muscle	↑ Insulin sensitivity, ↑ GLUT4-mediated glucose uptake, ↓ inflammation (GPCR120), ↑ GLP-1 & insulin secretion; in obesity/T2DM: ↓ PAHSAs, linked to insulin resistance & adipose inflammation.
Muscle/ adipocyte derived Exosomes/ miRNAs	Exosomes, miR-193a-5p, miR-99b, exoadipokines,	Adipose, fascia	Mediate inter-tissue crosstalk via miRNAs/proteins, regulate lipolysis, insulin sensitivity, ROS–NF-κB angiogenesis; in obesity/T2DM: ↑ Pro-inflammatory adipocyte exosomes (e.g., miR-193a-5p, damaged mitochondria) → insulin resistance, lipid dysregulation.
Other cytokines	TNF-α	Adipose, muscle, bone	Inhibits insulin signaling (adipose), ↓ FAO (muscle), ↑ osteoclastogenesis, ↓ osteoblasts via NF-κB/MAPK; in obesity/T2DM: ↑ TNF-α (VAT > SAT), ER stress-induced, → insulin resistance, bone lipid dysregulation.
	IL-1β	Adipose, muscle, bone	Impairs insulin signaling (muscle, adipose), ↑ osteoclastogenesis via NF-κB; in obesity/T2DM: ↑ IL-1β in inflamed WAT (macrophage-derived), → local insulin resistance, ↑ bone resorption.
Fibro-Adipogenic Mediators	PDGFRα+, thrombospondin-1, mast cell heparin	Fascia, muscle, adipose	Support ECM and regeneration, → adipogenesis/fibrosis under stress, ↑ lipid accumulation, ↓ insulin sensitivity, ↑ basal lipolysis, ↓ catecholamine response, secrete adipokines; in obesity/T2DM: ↑ FAP proliferation/adipogenesis, ↑ collagen/fibrosis, ↑ ectopic lipids, → fascia–muscle–adipose dysfunction, ↑ local insulin resistance.

* 3-HIB, 3-hydroxyisobutyrate; AMPK, AMP-activated protein kinase; aPKCλ/ζ, atypical protein kinase C lambda/zeta; BAIBA, β-aminoisobutyric acid; Cer, ceramides; CV, cardiovascular; DAG, diacylglycerol; ECM, extracellular matrix; ER, endoplasmic reticulum; FABP4, fatty acid–binding protein 4; FA, fatty acid; FAO, fatty acid oxidation; FAP, fibro-adipogenic progenitor; FGF, fibroblast growth factor; GLP-1, glucagon-like peptide-1; GLUT4, glucose transporter type 4; GPCR, G protein-coupled receptor; GPRC6A, G protein-coupled receptor class C group 6 member A; IL, interleukin; IMCL, intramyocellular lipid; IRS1, insulin receptor substrate 1; JAK, Janus kinase; LepR, leptin receptor; MAPK, mitogen-activated protein kinase; miRNA, microRNA; MOTS-c, mitochondrial open-reading frame of the 12S rRNA-c; NF-κB, nuclear factor kappa-light-chain-enhancer of activated B cells; nPKC, novel protein kinase C; OCN, osteocalcin; PAHSAs, palmitic acid esters of hydroxystearic acid; PGC1α, peroxisome proliferator-activated receptor gamma coactivator 1-alpha; PI3K, phosphoinositide 3-kinase; PKA, protein kinase A; PKCθ/ε/δ, protein kinase C theta/epsilon/delta; PP2A, protein phosphatase 2A; PPAR, peroxisome proliferator-activated receptor; RANKL, receptor activator of nuclear factor kappa-B ligand; ROS, reactive oxygen species; SAT, subcutaneous adipose tissue; SOST, sclerostin; STAT3, signal transducer and activator of transcription 3; T2DM, type 2 diabetes mellitus; TNF-α, tumor necrosis factor-alpha; UCP1, uncoupling protein 1; VAT, visceral adipose tissue; WAT, white adipose tissue.
